# Ganoderic acids-rich ethanol extract from *Ganoderma lucidum* protects against alcoholic liver injury and modulates intestinal microbiota in mice with excessive alcohol intake

**DOI:** 10.1016/j.crfs.2022.02.013

**Published:** 2022-02-24

**Authors:** Wei-Ling Guo, Ying-Jia Cao, Shi-Ze You, Qi Wu, Fang Zhang, Jin-Zhi Han, Xu-Cong Lv, Ping-Fan Rao, Lian-Zhong Ai, Li Ni

**Affiliations:** a Institute of Food Science and Technology, College of Biological Science and Technology, Fuzhou University, Fuzhou, Fujian, 350108, China; b School of Clinical Medicine, Fujian Medical University, Fuzhou, Fujian, 350122, China; c National Engineering Research Center of JUNCAO Technology, Fujian Agriculture and Forestry University, Fuzhou, Fujian, 350002, China; d School of Medical Instruments and Food Engineering, University of Shanghai for Science and Technology, Shanghai, 200093, China; e School of Food Science and Technology, Jiangnan University, Wuxi, Jiangsu, 214122, China

**Keywords:** *Ganoderma lucidum*, Triterpenoids, alcoholic liver injury, Intestinal microbiota, Liver metabolomics

## Abstract

Alcoholic liver injury is mainly caused by excessive alcohol consumption and has become a global public health problem threatening human health. It is well known that *Ganoderma lucidum* possesses various excellent beneficial effects on liver function and lipid metabolism. The purpose of this study was to evaluate the underlying protective effect and action mechanism of ganoderic acids-rich *G. lucidum* ethanol extract (GLE) on alcohol-induced liver injury in mice with excessive alcohol intake. Results showed that oral administration of GLE could obviously inhibit the abnormal increases of serum triglyceride (TG), total cholesterol (TC), low density lipoprotein cholesterol (LDL-C), aspartate aminotransferase (AST) and alanine aminotransferase (ALT), and also significantly protect the liver against alcohol-induced excessive hepatic lipid accumulation and pathological changes. In addition, alcohol-induced oxidative stress in liver was significantly ameliorated by the dietary intervention of GLE through reducing the hepatic levels of maleic dialdehyde (MDA) and lactate dehydrogenase (LDH), and increasing the hepatic levels of glutathione (GSH), catalase (CAT), superoxide dismutase (SOD) and alcohol dehydrogenase (ADH). Compared with the model group, GLE intervention significantly ameliorated the intestinal microbial disorder by elevating the relative abundance of *Ruminiclostridium*_9, *Prevotellaceae*_UCG-001, *Oscillibacter*, *[Eubacterium]_xylanophilum*_group, norank_f_Clostridiates_vadinBB60_group, GCA-900066225, *Bilophila*, *Ruminococcaceae_*UCG-009, norank_f_*Desulfovibrionaceae* and *Hydrogenoanaerobacterium*, but decreasing the proportion of *Clostridium*_sensu_*stricto*_1. Furthermore, liver metabolomic profiling suggested that GLE intervention had a significant regulatory effect on the composition of liver metabolites in mice with excessive alcohol intake, especially the levels of some biomarkers involved in primary bile acid biosynthesis, riboflavin metabolism, tryptophan metabolism, biosynthesis of unsaturated fatty acids, fructose and mannose metabolism, glycolysis/gluconeogenesis. Additionally, dietary supplementation with GLE significantly regulated the mRNA levels of key genes related to fatty acids metabolism, ethanol catabolism and inflammatory response in liver. Conclusively, these findings indicate that GLE has a potentially beneficial effect on alleviating alcohol-induced liver injury and may be developed as a promising functional food ingredient.

## Highlights

• Phytochemical analysis revealed that ethanol extract of *Gaoderma lucidum* (GLE) is rich in ganoderic acids.
• GLE ameliorated lipid metabolism, antioxidant function and inflammatory response in mice with excessive alcohol intake.
• Liver metabolomics based on UPLC-QTOF/MS was performed to reveal the underlying hepatoprotective effect of GLE.
• GLE intervention alleviated alcoholic liver injury partly through regulating the “gut-liver-metabolite”axis.
• Hepatic gene transcriptions related to lipid metabolism and inflammation were remarkablyinfluenced by GLE intervention.

## Graphical abstract

The beneficial effects of *Ganoderma lucidum* ethanol extract (GLE) on liver metabolism function and intestinal microbiota in mice with excessive alcohol intake.

**Figure:**
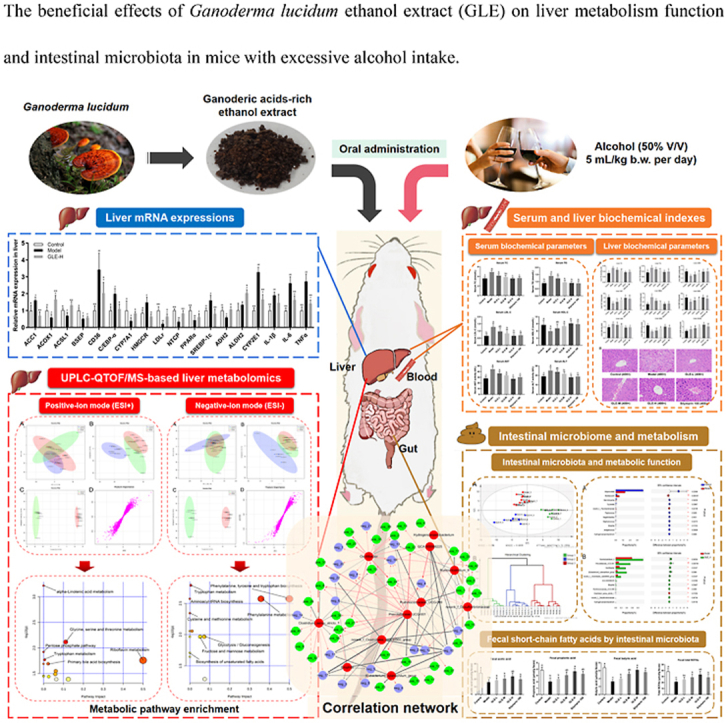

## 1 Introduction

Alcoholic beverage is one of the most popular drink that consumed by billions of people worldwide (Spear, 2018). However, excessive alcohol intake will destroy the liver metabolic function and the homeostasis of oxidative stress, and thereby adversely affecting the human health (Rungratanawanich et al., 2021). Alcoholic liver injury is one of the most prevalent liver diseases caused by excessive alcohol intake, which has attracted wide attention due to the high morbidity and mortality worldwide. It is well known that excessive alcohol consumption produces a large number of destructive endogenous free radicals, which are difficult to remove from the body in a short time, leading to liver damage, steatohepatitis, fibrosis, cirrhosis, hepatocyte necrosis and even liver failure (Li et al., 2021). Alcoholic liver injury is mainly characterized by hepatic steatosis and inflammation (Seitz et al., 2018), and patients with alcoholic liver injury usually suffer from hyperlipidemia, hyperglycemia and metabolic inflammation (Jakkaew et al., 2019). Drug therapy is considered as the current primary treatment for alcoholic liver injury, while most therapeutic drugs still have a series of unpleasant side effects (Tijera et al., 2014; Watanabe et al., 2017). Therefore, looking for natural products with strong liver protective effect from natural food resources is a promising strategy to intervene the pathological development of alcoholic liver injury.

*Ganoderma lucidum* (*Lingzhi*) is one of the most popular and well-known mushroom of “medicine and food homology” in China, and has been widely applied as a traditional medicine in Eastern countries for thousands of years. Accumulating evidence demonstrated that *G. lucidum* possess a broad of nutrients and bioactive ingredients, including polysaccharides, proteins, polysaccharide-peptides, alkaloids, phenolic acids and triterpenoids, etc (Chang et al., 2015; Lv et al., 2019). Among them, triterpenoids are the most important chemical components in *G. lucidum* fruiting bodies and have been proved to possess a variety of pharmacological activities, including anti-cancer, anti-oxidant, immunomodulatory, hypoglycemic and hypolipidemic properties (Ahmad et al., 2021). Our previous study showed that *G. lucidum* ethanol extract (GLE) is mainly consisted of ganoderic acid A, ganoderic acid η, poricoic acid HM, ganoderic acid Me, 12-hydroxyganoderic acid C2, and so on (Guo et al., 2018). It has been previously revealed that oral administration of GLE ameliorated hyperlipidemia in rats fed with a high-fat diet by regulating the mRNA expression of key genes related to lipid and cholesterol metabolism (Guo et al., 2018; Zhu et al., 2018). Moreover, it was also reported that GLE protected against oxidative stress and liver pathological process, and thus may be useful in the management of metabolic syndrome and its complications (Li et al., 2021; Chang et al., 2015; Guo et al., 2018; Adeyi et al., 2021). It is well known that GLE possesses excellent hypolipidemic and hepatoprotective effects, providing a potential viable regimen in the prevention or alleviation of alcoholic liver injury. Furthermore, despite the reported beneficial effects of GLE on liver, whether GLE administration can protect against the pathological process of alcoholic liver injury and its potential mechanism of action have not yet been fully elucidated.

The “gut-liver-metabolite” axis plays an important role in the pathological development of alcoholic liver injury (Ray, 2017). Accumulating evidences suggest that excessive alcohol intake may cause the disorders of intestinal microbial homeostasis and the permeability of intestinal barrier, resulting in the proliferation of harmful bacteria in the intestinal tract, the absorption of harmful metabolites, and the damage of liver metabolism function (Tian et al., 2020). Previous study had shown that the intestines of alcoholics are characterized by lower abundance of *Eisenbergiella* and *Akkermansia*, but higher proportion of *Odoribacter*, *Parasutterella* and *Psychrobacter* (Sang et al., 2021; Song et al., 2021; Cao et al., 2021). According to the previous report, *Bifidobacterium* and *Akkermansia* could significantly improve the intestinal barrier function and reduce the damage caused by alcohol metabolites (acetaldehyde and free radicals) to the liver in mice with excessive alcohol intake (Bajaj, 2019). Besides, excessive alcohol consumption also significantly changes the metabolites produced by intestinal microbiota, such as short-chain fatty acids (SCFAs) and bile acids (BAs) (Schnabl and Brenner, 2014). It is widely accepted that the intestinal SCFAs level is highly linked to the structure of intestinal microbiota, the functions of liver metabolism and insulin resistance (Shimizu et al., 2019). Therefore, modulating the intestinal microbiota by probiotics or bio-active natural products, especially promoting the proliferation of intestinal probiotics and inhibiting the growth/metabolism of harmful bacteria, would be a potential effective approach to prevent or treat alcoholic liver injury (Ray, 2017). So far, few studies have attempted to describe the beneficial effects of GLE on the composition of the intestinal flora and its association with the hepatoprotective effects in mice with excessive alcohol intake.

The purpose of this research was to investigate the protective effect and underlying action mechanism of GLE against alcoholic liver injury in mice with excessive alcohol intake, through high-throughput sequencing and liver metabolomics coupled with metabolic pathway analysis. Moreover, the connections between the key intestinal microbial phylotypes and liver biomarkers were also revealed through correlation analysis and visualized by network, providing a strong theoretical basis for developing functional foods to improve alcoholic liver injury.

## 2 Materials and methods

### 2.1 Materials and chemical reagents

Ethanol extract of *G. lucidum* (GLE) was prepared from the fruiting body of *G. lucidum* (provided by National Engineering Research Center of JUNCAO Technology, Fujian Agriculture and Forestry University) according to our previous study (Guo et al., 2018). Briefly, the fruiting body of *G. lucidum* was dried in an oven at 70 °C for 24 h, and fine powder was obtained by grinding with an ultra-fine pulverizer and passing through a 60-mesh sieve. The powder was soaked using 95% ethanol (v/v) at 60 °C for 2 h (solid-liquid ratio of 1:20). The supernatant was collected by 8-layer gauze filtration and low-temperature centrifugation (5000 g at 4 °C for 20 min). The filtered residue was extracted twice. The obtained supernatant was combined and concentrated under reduced pressure at 50 °C, in order to remove the water and ethanol. Finally, the concentrated samples were freeze-dried for further exploration. Compound composition of GLE was determined through high performance liquid chromatography (Agilent 1260, CA, USA) coupled with QTOF electrospray ionization MS system (Agilent 6530, CA, USA) (Supplementary Materials). The commercial test kits for the detections of total cholesterol (TC), triglyceride (TG), low-density lipoprotein cholesterol (LDL-C), high-density lipoprotein cholesterol (HDL-C), aspartate transaminase (AST), alanine transaminase (ALT), glutathione (GSH), catalase (CAT), maleic dialdehyde (MDA), superoxide dismutase (SOD), lactate dehydrogenase (LDH), alcohol dehydrogenase (ADH) and aldehyde dehydrogenase (ALDH) were purchased from Nanjing Jiancheng Biotech Co., Ltd. (Nanjing, China). Other chemical reagents used in this study are analytical grade reagents, which were purchased from Shanghai Sangon Biotech Co., Ltd. (Shanghai, China).).

### 2.2 Animal experimental design

Fifty specific pathogen-free male Kunming mice (6–7 weeks age, 18–22 g body weight) were obtained from the Animal Center of Fujian Medical University (Fuzhou, China). All mice were housed in a SPF grade animal laboratory (temperature: 24 ± 1 °C, relative humidity: 60 ± 5%, light-dark cycle: 12 h). All mice were given free access to diet and water. After 7 days of acclimatization, all mice were assigned into five groups: the Control group (n = 8); the Model group (n = 10); the GLE-L group (n = 8, oral gavage with low-dose GLE [25 mg/kg b.w.]); the GLE-M group (n = 8, oral gavage with medium-dose GLE [50 mg/kg b.w.]); the GLE-H group (n = 8, oral gavage with high-dose GLE [100 mg/kg b.w.]); the Silymarin 100 group (positive control group, n = 8, oral gavage with silymarin [100 mg/kg b.w.]). Mice in the GLE-L, GLE-M, GLE-H groups or silymarin 100 group were gavaged with 200 μL GLE or silymarin suspension (the solvent was 0.5% CMC-Na), respectively, and the mice in the control group and model group were gavaged with the same volume of 0.5% CMC-Na instead. Mice of the Model, GLE-L, GLE-M, GLE-H and Silymarin 100 groups were intragastrically administered with 50% alcohol solution (v/v, 5 mL/kg b.w. every day) 4 h after the administration of GLE or silymarin every day, while mice of Control group were only received equal amount of physiological saline. The body weight of each mouse was recorded every week in the experimental period. After 6 weeks of intervention, all mice were fasted for 12 h and euthanized under anesthesia. Fresh blood and cecal contents were collected from each mouse and put into 2.0 mL sterile tubes. Blood samples were stored at room temperature for 0.5 h and centrifuged for 10 min to obtain serum and stored at −20 °C. Liver samples were weighed immediately and then frozen in liquid nitrogen and finally maintained at an ultra cold storage freezer (−80 °C) until further analysis. The animal experimental protocols were conducted in accordance with the guidelines of the Laboratory Animal Welfare and approved by the Animal Ethics Committee of Institute of Food Science and Technology, Fuzhou University, China (No.: FZU-FST-2021-006).

### 2.3 Biochemical assays of the serum and liver samples

Serum concentrations of TC, TG, LDL-C, HDL-C, AST and ALT were measured with the blood automatic biochemical analyzer (Toshiba TBA-40FR, Japan) according to the operating instructions provided by the commercial kits. A small piece of liver sample of each mouse was put into physiological saline and homogenized by a high-speed homogenizer to obtain liver homogenate. The supernatants were collected by centrifugation at 3000*g* and 4 °C for 10 min. The levels of hepatic TC, TG, GSH, CAT, MDA, SOD, LDH, ADH and ALDH were determined using commercial kits according to instructions (Jiancheng Bio. Ins., Nanjing, China).

### 2.4 Hematoxylin–eosin (H&E) staining

For histopathological evaluation, fresh liver sections were fixed in 4% paraformaldehyde for 24 h following by dehydration through a series of ethanol solutions, and embedded in paraffin and cut into section (5 μm thickness). After the fabricated liver sections were stained with H&E, they were photographed under light microscope (Nikon, Japan).

### 2.5 Measurement of short chain fatty acids (SCFAs)

SCFAs in fecal samples were extracted and determined according to our previously reported method with appropriate modifications (Guo et al., 2020a). Briefly, saturated NaCl solution (500 μL) was added to dried feces (50 mg) and placed at room temperature (25 °C) for 0.5 h, followed by homogenization on a high-speed homogenizer for 3 min. Then, 20 μL H_2_SO_4_ (10%, v/v) was added and mixed with a vortex for 30 s. The total SCFAs were completely collected with 800 μL anhydrous ether and then centrifuged (10,000 g, 10 min, 4 °C). Finally, the residual trace water in the supernatants were removed with anhydrous Na_2_SO_4_, and the contents of SCFAs in the supernatants were determined by Agilent 7890B gas chromatography system equipped with Agilent J&W DB-WAX capillary column (30 m × 0.25 mm × 0.25 μm) and flame ionization detector.

### 2.6 High throughput sequencing and bioinformatics analysis

Bacterial DNAs were extracted from cecal content samples using genomic DNA extraction kit (Omega, USA) and quantified by NanoDrop Spectrophotometer (Thermo Fisher Scientific). The V3–V4 hypervariable regions of 16S rDNA sequence were sequenced by high throughput sequencing based on Illumina MiSeq platform at Shanghai Majorbio Co., Ltd. (Shanghai, China) according to our previous study (Guo et al., 2020a). The sequencing raw data were imported into QIIME 2 software, and the filtered sequences were clustered into operation taxon units (OTU) with 97% identity threshold. Based on the GreenGenes database (Ver. 13.8), the sequence similarity was matched to identify microbial phylotypes at the genus level, and the relative abundance of each OTU was obtained. Principal component analysis (PCA) and hierarchical clustering were implemented to assessed the intestinal microbial composition by SIMCA (Ver. 15.0). Differences between different experimental groups were conducted using linear discriminant analysis coupled with effect size (LEfSe) based on Galaxy Online Platform (http://huttenhowe.sph.harvard.edu/galaxy/). PICRUSt 2.0 was applied to forecast the abundances of functional gene categories on KEGG pathway database. The correlations of the key intestinal bacterial phylotypes with the liver metabolites (liver biomarkers) significantly regulated by GLE intervention were revealed by correlation analysis through R software (Ver. 3.3.3) and visualized by network through Cytoscape (Ver. 3.6.0).

### 2.7 Untargeted liver metabolomics analysis

Liver sections (25 mg) were extracted at the mixed organic solvent (acetonitrile: methanol: water = 2: 2: 1), and vibrated on a vortex oscillator for 1 min. After centrifugation at 10,000*g* for 15 min at 4 °C, the supernatant was taken into a centrifuge tube and evaporated to dryness at 37 °C under a gentle stream of nitrogen. The dried samples were reconstituted in 200 μL of 50% acetonitrile, and centrifugated at 12,000 g for 10 min at 4 °C. The supernatant fluid was injected into ultra-performance liquid chromatography-quadrupole time-of-flight mass spectrometry (UPLC-QTOF/MS) system for metabolomics analysis. UPLC separation of liver metabolites was carried out on Agilent 1290 Infinity UPLC equipped with BEH Amide column (2.1 mm × 100 mm, 1.7 μm, Waters) and 6530 QTOF electrospray ionization MS (Agilent, CA, USA).

Raw data obtained from UPLC-QTOF/MS system were converted and analyzed by MPP software (Agilent, CA, USA) for peak detection, alignment and identification. The peak intensities were exported to SIMCA 15.0 software for multivariate statistical analysis including PCA, PLS-DA and OPLS-DA. Liver metabolites of significant difference between the model and GLE-H groups were selected by OPLS-DA (VIP value > 1.0, *p* < 0.05). The selected metabolites were then identified with HMDB database. Pathway analysis of liver metabolites with significant difference was performed on the online analysis platform MetaboAnalyst 5.0.

### 2.8 Reverse transcription-quantitative polymerase chain reaction (RT-qPCR)

Total RNA was extracted by a commercial RNA extraction kit [RNAiso Plus, Code No. 9108] provided by Takara Biomedical Technology (Beijing) Co., Ltd., and then reverse-transcribed into cDNA using a commercial cDNA kit with gDNA Eraser [Code No. RR047A] (Takara, Beijing, China). qPCR was completed in StepOne Plus Real-Time quantitative PCR System (Applied Biosystems, Foster City, CA, USA) with SYBE Green Ex Taq™ II [Tli RNaseH Plus, Code No. RR820A] (Takara, Beijing, China). The PCR conditions were as following: initial activation 95 °C for 30s, denaturation 95 °C for 5s, annealing 55 °C for 30 s, extension 72 °C for 30 s, 40 cycles. The mRNA expressions level was normalized to 18S rDNA gene. The 2^−ΔΔCT^ standard method was used for analyzing the data obtained from real-time qPCR. The qPCR primers used in this study were listed in Table 1.

**Table 1:** Primer sequences for quantitative real-time PCR.

Gene	Forward primer (5′–3′)	Reverse primer (5′–3′)
*ADH2*	AACGGTGAGAAGTTCCCAAAA	ACGACCCCCAGCCTAATACA
*ALDH2*	ATCCTCGGCTACATCAAATCG	GTCTTTTACGTCCCCGAACAC
*CYP2E1*	CCAACTCTGGACTCCCTTTTAT	ACGCCTTGAAATAGTCACTGTA
*IL-1β*	GCAACTGTTCCTGAACTCAACT	ATCTTTTGGGGTCCGTCAACT
*IL-6*	TTCTCTGGGAAATCGTGGAAA	TGCAAGTGCATCATCGTTGT
*TNFα*	CCAGTGTGGGAAGCTGTCTT	AAGCAAAAGAGGAGGCAACA
*IFN-γ*	ACAGCAAGGCGAAAAAGGATG	TGGTGGACCACTCGGATGA
*ACC1*	GCCATCCGGTTTGTTGTCA	GGATACCTGCAGTTTGAGCCA
*ACOX1*	GCCTGCTGTGTGGGTATGTCATT	GTCATGGGCGGGTGCAT
*ACSL1*	CACTTCTTGCCTCGTTCCAC	GTCGTCCCGCTCTATGACAC
*BSEP*	TCTGACTCAGTGATTCTTCGCA	CCCATAAACATCAGCCAGTTGT
*CD36*	ACTTGGGATTGGAGTGGTGATGT	GGATACCTGCAGTTTGAGCCA
*C/EBP-α*	GAACAGCAACGAGTACCGGGTA	GCCATGGCCTTGACCAAGGAG
*CYP7A1*	CCTTGGGACGTTTTCCTGCT	GCGCTCTTTGATTTAGGAAG
*HMGCR*	TGCTGGTGCTATCAAAGG	GCAGATGGGATGACTCGA
*LDLr*	ATGCTGGAGATAGAGTGGAGTT	CCGCCAAGATCAAGAAAG
*NTCP*	CAAACCTCAGAAGGACCAAACA	GTAGGAGGATTATTCCCGTTGTG
*PPARα*	CCTGGAAAGTCCCTTATCT	GCCCTTACAGCCTTCACAT
*SREBP-1c*	GCCGGCGCCATGGACGAGCTGG	CAGGAAGGCTTCCAGAGAGGAG
Mouse 18S	AGTCCCTGCCCTTTGTACACA	CGATCCCAGGGCCTCACTA

### 2.9 Statistical analysis

All data of this study were indicated as the mean ± standard deviation. The statistical significances of results were analyzed by one-way analysis of variance (ANOVA) base on Duncan's multiple range test with GraphPad Prism 7.0. The significance level in the analyses was considered *p* < 0.05.

## 3 Results and discussion

### 3.1 HPLC-QTOF/MS analysis of the compound composition of GLE

Phytochemical analysis of *G. lucidum* ethanol extract (GLE) was performed through HPLC-QTOF/MS technique. In the current study, the negative ionization mode was preferred because of the higher sensitivity and selectivity for the detection of triterpenes or ganoderic acids, as these compounds have one or more hydroxyl and/or carboxylic acid groups. The total ion chromatogram showed that GLE mainly contains eighteen compounds (Fig. S1, the top 18 according to peak area percentage), which were then tentatively identified by retention times, pseudomolecular ions and their ion-fragmentation patterns observed in MS2 spectra. The information (accurate mass of the pseudomolecular ions for the peaks and their fragmentation pattern) provided by reference standards and from the previously reported literatures were also employed for the identification of the eighteen compounds in GLE (Yang et al., 2007; Chen et al., 2019; Guo et al., 2012). The retention times and mass spectrum data along with the peak assignments for compounds identified using negative ionization are described in Table 2. A total of eighteen triterpenoids were finally identified in GLE, among which ganoderic acid B, ganoderic acid A, ganoderic acid H, ganoderic acid D, 12-acetoxyganoderic acid F were the representatives with high peak area percentage.

**Table 2:** Characteristics of sixteen peaks from the GLE identified by HPLC-MS/MS in the negative ionization mode.

Peak No.	Rt (min)	UV *λ*_max_ (nm)	Molecular formula	Tentative identification	[M-H]- (m/z)	Fragmentation pattern (MS2, m/z)	Reference
1	10.237	256	C_30_H_40_O_8_	Elfvingic acid A	527.2595	509.2453, 465.2562, 421.2668, 317.1699	Yang et al. (2007)
2	14.850	255	C_30_H_44_O_8_	Ganoderic acid η	531.2866	513.2777,129.0527,111.0425	Yang et al. (2007)
3	18.933	254	C_30_H_44_O_8_	Ganoderic acid G	531.2929	513.2783, 469.2913, 451.2769, 436.2610, 319.1892, 265.1389, 249.1467	Yang et al. (2007)
4	21.751	254	C_30_H_38_O_8_	Ganosporeric acid A	525.2464	507.2344, 451.2106, 129.0529, 495.1996, 229.1176	Chen et al. (2019)
5	23.523	256	C_30_H_42_O_8_	Ganodoeric acid C6	529.2715	511.2592, 493.2432, 481.2115, 467.2702, 449.2608, 437.2232, 317.1724, 303.1524	Yang et al. (2007)
6	26.600	249	C_30_H_42_O_7_	Ganoderenic acid B	513.2810	451.2839, 436.2592, 287.1642, 249.1462	Yang et al. (2007)
7	27.501	254	C_30_H_42_O_7_	Ganoderic acid Xi	513.2764	495.2726, 465.2214, 451.2825, 383.2162, 331.1900, 235.1690, 151.1109, 73.0285	Chen et al. (2019)
8	29.497	253	C_30_H_44_O_7_	Ganoderic acid B	515.2975	497.2840, 453.2940, 438.2717, 420.2620, 303.1926, 263.1626, 249.1457, 195.1362	Yang et al. (2007)
9	33.993	256	C_32_H_46_O_9_	Ganoderic acid K	573.3030	555.2904, 511.2988, 469.2920, 451.2807, 302.1843, 265.1405	Yang et al. (2007)
10	37.460	253	C_30_H_44_O_7_	Ganoderic acid A	515.2923	497.2807, 453.2919, 435.2819, 195.0978	Yang et al. (2007)
11	38.257	254	C_32_H_44_O_9_	Ganoderic acid H	571.2862	553.2768, 511.2671, 467.2768, 437.2306, 423.2668, 303.1578	Yang et al. (2007)
12	41.500	245	C_30_H_40_O_8_	Ganoderic acid derivative	527.6340	n.d	Yang et al. (2007)
13	42.840	254	C_30_H_40_O_7_	Ganoderic acid E	511.2671	493.2484, 449.2638, 434.2381, 285.1442	Guo et al. (2012)
14	44.873	256	C_30_H_42_O_8_	12-Hydroxyganoderic acid D	529.2702	511.2629, 493.2510, 449.2618, 434.2406, 301.1764	Yang et al. (2007)
15	47.163	256	C_30_H_42_O_7_	Ganoderic acid D	513.2782	495.2657, 451.2789, 301.1766, 283.1649, 247.1302, 193.1199	Yang et al. (2007)
16	50.530	260	C_32_H_42_O_9_	Ganoderic acid F	511.2602	493.2537, 449.2638, 434.2404, 247.1307, 149.0509	Yang et al. (2007)
17	54.680	250	C_32_H_42_O_9_	12-Acetoxyganoderic acid F	569.2656	511.2525, 509.2514, 479.2054, 465.2617, 435.2144	Chen et al. (2019)
18	58.012	254	C_30_H_42_O_7_	Ganoderic acid J	513.2852	495.2747, 469.2954, 451.2848, 409.2378	Yang et al. (2007)

### 3.2 Effects of GLE on body growth performance in mice with excessive alcohol intake

**Fig. 1:**
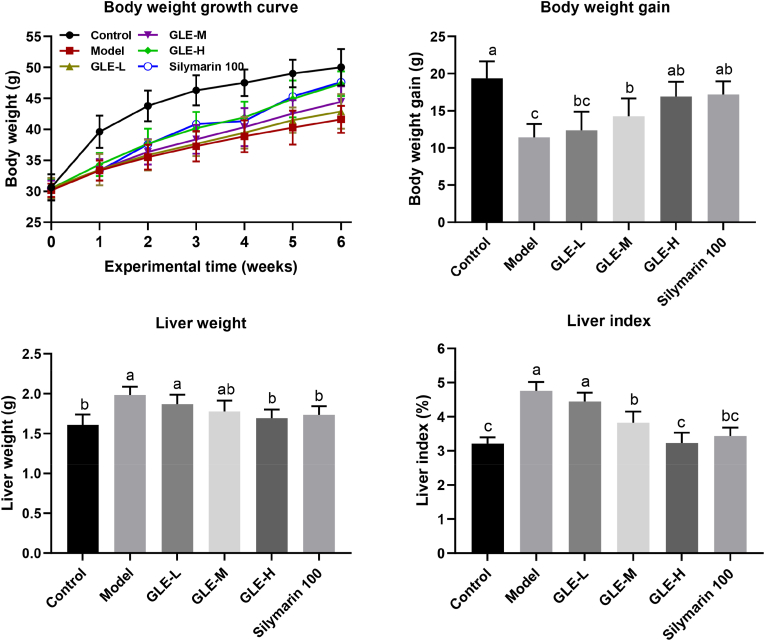
Effects of GLE intervention on the body weight, liver weight and liver index in mice with excessive alcohol intake. Values were expressed as mean ± SEM (n = 8), and different letters represent significant differences between different experimental groups (*p* < 0.05).

As shown in Fig. 1, there was no significant difference in the body weight of mice among different experimental groups at the beginning of the experiment. However, after 6 weeks' intragastric administration of alcohol, the body weight of mice in the Model group were significantly lower than that of the Control group (*p* < 0.05), indicating that excessive alcohol intake may damage the body's metabolic function. Oral administration of GLE significantly reversed the abnormal reduction of body weight induced by excessive alcohol intake in a dose-dependent manner. The liver plays a crucial role in regulating the alcohol metabolism because more than 80% alcohol is consumed in the liver (Stornetta et al., 2018). Liver index is one of the most sensitive indicators of alcohol metabolism, and can effectively reflect the degree of liver injury to a certain extent. In this study, the liver weight and liver index of the Model group were significantly higher than those of the Control group (*p* < 0.05), suggesting that excessive alcohol intake may cause liver tissue injury in mice. It has been well known that silymarin had a protective effect on the liver function. There was no significant difference in liver index between the mice in the Control group and the Silymarin 100 group. Compared with mice of the Model group, oral administration of GLE at 50 and 100 mg/kg b.w. significantly reduced the liver index of mice with excessive alcohol intake (*p* < 0.05), suggesting GLE could effectively protect against alcohol-induced liver injury.

### 3.3 Effects of GLE intervention on serum biochemical parameters

**Fig. 2:**
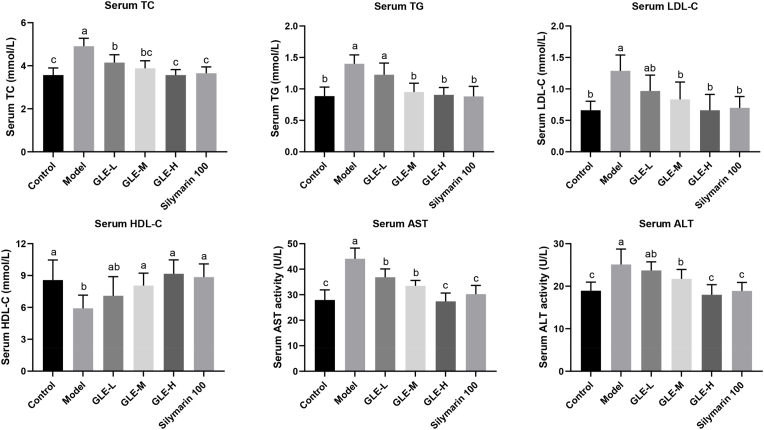
Effects of GLE intervention on the serum biochemical parameters (TC, TG, LDL-C, HDL-C, AST, and ALT) in mice with excessive alcohol intake. Values were expressed as mean ± SEM (n = 8), and different letters represent significant differences between different experimental groups (*p* < 0.05).

The abnormal increase of serum TC, TG, LDL-C, AST, ALT levels, and the abnormal decrease of serum HDL-C levels are the main characteristics in mice with alcoholic liver injury (Do et al., 2021). In this study, excessive alcohol consumption markedly increased the serum TC, TG, LDL-C, AST, ALT levels (*p* < 0.05), and significantly reduced the serum HDL-C level (*p* < 0.05), indicating the liver metabolic function was destroyed in the mice with excessive alcohol intake (Fig. 2). Serum LDL-C and TG levels are generally considered to be key indicators closely related to lipid metabolism diseases. Therefore, inhibiting the abnormal increase of serum LDL-C and TG levels is beneficial to reduce the risk of lipid metabolism disorder. After six weeks of GLE administration, the serum levels of TC, TG and LDL-C were significantly decreased compared with those in the model group (*p* < 0.05), while the serum HDL-C level was significantly increased, especially in mice with oral supplementation of high-dose GLE (100 mg/kg b.w.), which is comparable to equivalent dose of silymarin (100 mg/kg b.w.). Moreover, as the two most important and sensitive indicators of liver metabolic function, serum ALT and AST levels were also significantly increased in mice exposed to alcohol intake (*p* < 0.05), reflecting the existence of liver inflammation or liver injury (El-Newary et al., 2017). Interestingly, oral supplementation of medium-dose and high-dose GLE (50 and 100 mg/kg b.w.) significantly down-regulated alcohol-induced abnormal increase in serum ALT and AST levels. Taken together, these results suggested that alcohol-induced liver injury can be effectively attenuated by daily administration with GLE in a dose-dependent manner.

### 3.4 Effects of GLE on liver biochemical parameters and histopathological features

**Fig. 3:**
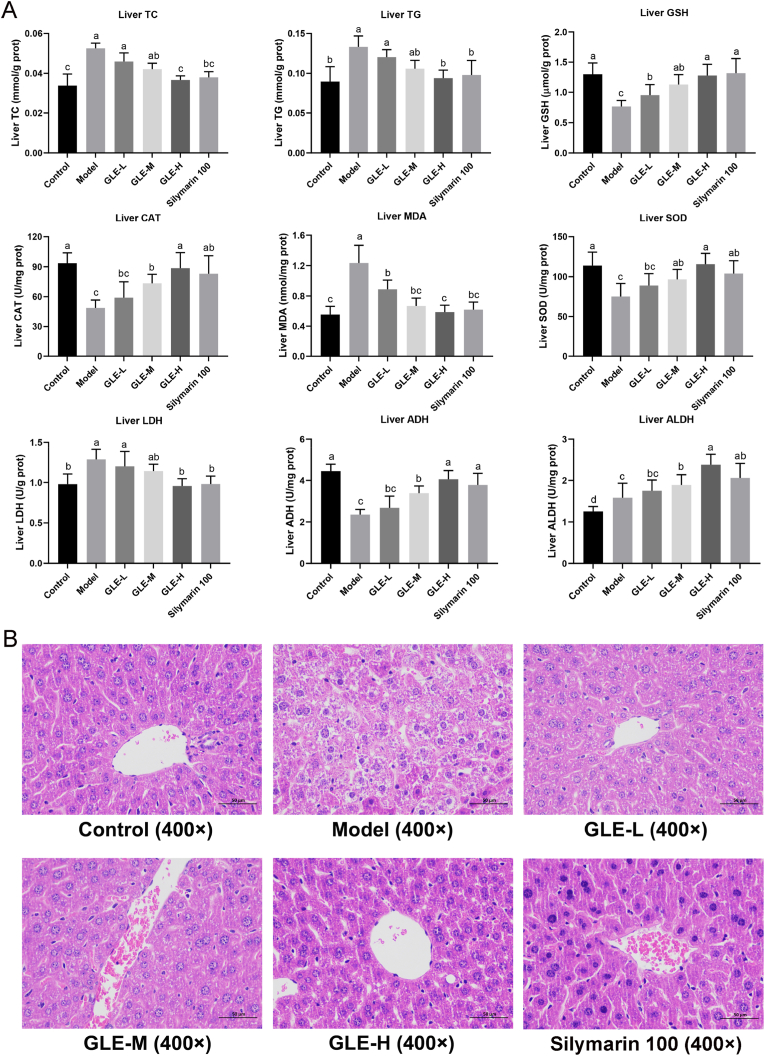
Effects of GLE intervention on the liver biochemical parameters (TC, TG, GSH, CAT, MDA, SOD, LDH, ADH and ALDH) and liver histopathological features in mice with excessive alcohol consumption. Values were expressed as mean ± SEM (n = 8), and different letters represent significant differences between different experimental groups (*p* < 0.05).

It is well known that the liver is a crucial organ closely related to alcohol metabolism. It can convert alcohol into acetaldehyde, which in turn can be converted into acetic acid. However, excessive alcohol consumption may lead to the metabolic dysfunction of liver, which is characterized by higher levels of liver lipids and oxidative stress, and lower activities of enzymes related to alcohol metabolism (Han et al., 2021). As depicted in Fig. 3A, the mice with excessive alcohol intake (the Model group) were characterized by higher levels of liver TC and TG, suggesting that excessive alcohol intake may destroy the function of liver lipid metabolism. Compared with the Model group, the levels of TC and TG in liver were obviously ameliorated with GLE intervention, especially in mice with high-dose GLE intervention (100 mg/kg b.w.), which were similar to those in Control group (*p* > 0.05). The effects of high-dose GLE intervention on liver TC and TG were comparable to that of silymarin, indicating that GLE can provide anti-hyperlipidemic activity against alcohol toxicity.

Oxidative stress status reflects the degree of liver injury induced by excessive alcohol consumption, thus the oxidative stress-related parameters including GSH, CAT, MDA, SOD and LDH in liver were also measured with assay kits (Hasan et al., 2020). It was previously reported that long-term alcohol consumption significantly elevated the level of liver MDA that is the product of lipid peroxidation, which may directly destroy the normal structure of hepatocytes (Qu et al., 2019). Compared with the Control group, the liver levels of GSH, CAT and SOD were significantly reduced, but MDA and LDH levels were obviously elevated in mice of the Model group (*p* < 0.05). It is well known that superoxide radicals are converted into hydrogen peroxide through the catalytic action of SOD, and then hydrogen peroxide is decomposed into to water and oxygen under the action of GSH and CAT. Therefore, elevating the activities of GSH, CAT and SOD in liver is helpful to reduce the production of reactive oxygen species caused by excessive alcohol intake, so as to prevent the pathological development of alcoholic liver injury (Hasan et al., 2020). In the present study, oral administration of GLE significantly elevated the activities of GSH, CAT and SOD in liver (*p* < 0.05), and decreased the hepatic level of MDA in a dose-dependent manner, suggesting that GLE intervention could relieve alcohol-induced liver injury by elevating the activities of antioxidant enzymes and inhibiting the oxidative stress in liver.

It is well established that ethanol is catalyzed by ADH and ALDH into acetaldehyde and non-toxic acetate in the liver, respectively. Excessive amounts of both can cause oxidative stress and apoptosis of liver cells, leading to liver tissue inflammation and fibrosis. Acetaldehyde accumulation may occur in the liver when excessive alcohol consumption impairs the activities of ADH and ALDH. Thus, activation of the activities of ADH and ALDH in liver is beneficial for reducing the risk of alcoholic liver injury. Our study showed that GLE intervention (especially high-dose GLE) significantly elevated the activities of ADH and ALDH in liver of mice with excessive alcohol intake (*p* < 0.05) (Fig. 3A).

Histopathological analysis of liver sections of mice in the different experimental groups were observed. As shown in Fig. 3B, the mice of the Control group had evident liver lobule, orderly arrangements of liver cell cords, round central nucleus, distinct cytoplasm and cell borders. However, excessive alcohol consumption induced hepatocyte turbid, cytoplasm pale and nucleus unclear, indicated that the accumulation of lipids in intracellular vesicles. Compared with the model group, GLE intervention obviously prevented alcohol-induced hepatocyte turbidity, swelling, foam like changes and cord like disorder in a dose-dependent manner. In mice with high-dose GLE intervention, hepatocyte turbidity swelling and foam-like cells were significantly reduced, similar to mice of the Control group without alcohol intake.

### 3.5 GLE intervention elevated the fecal SCFAs in mice with excessive alcohol intake

**Fig. 4:**
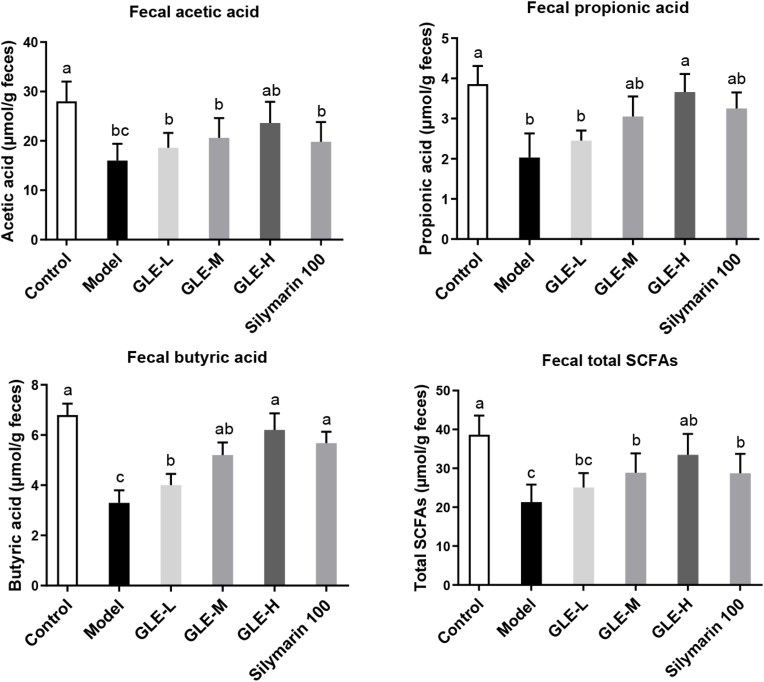
Effects of GLE intervention on the fecal short-chain fatty acids (SCFAs, including acetic acid, propionic acid and butyric acid) in mice with excessive alcohol consumption for consecutive 6 weeks. Values were expressed as mean ± SEM (n = 8), and ifferent letters represent significant differences between different experimental groups (*p* < 0.05).

Current evidence has confirmed that SCFAs play an important role in maintaining intestinal health, because they can be used as energy source for intestinal epithelial cells and regulating intestinal immune function (Yang et al., 2020). Elevation of intestinal SCFAs is benefit for maintaining the homeostasis of intestinal function, and thus preventing against the damage of liver function (Li et al., 2018). As shown in Fig. 4, compared with mice of the Control group, the fecal SCFAs, acetic acid, propionic acid and butyric acid levels were significantly reduced in mice with excessive alcohol intake (the Model group) (*p* < 0.01). Previous study had showed that long-term excessive alcohol consumption significantly reduced the fecal levels of SCFAs, especially in patients with alcoholic hepatitis, which is closely related to the reduction of the abundance of SCFAs producing bacteria in the intestine (Fairfield and Schnabl, 2021). Interestingly, high-dose GLE intervention (100 mg/kg b.w.) significantly enhanced the concentration of SCFAs (including acetic acid, propionic acid and butyric acid) in the gut (*p* < 0.05). It has been previously reported that the increase of acetic acid in the intestine greatly affected the metabolism of glycolipids and fatty acids, thus controlling the homeostasis of colonic Treg cells (Smith et al., 2013). It was also found that propionic acid could inhibit the biosynthesis of cholesterol and fatty acids in mouse liver (Liang et al., 2019), and improve social behavior disorders and cognitive functions impairment by crossing intestinal-blood and blood-brain barriers (Huang et al., 2021c). Butyric acid plays an important role in regulating the lipid metabolism and is helpful to decrease the risk of lipid metabolism disorder in liver (Skariyachan and Bs, 2021). Therefore, GLE intervention is likely to prevent alcohol-induced liver damage by regulating the SCFAs production of intestinal flora, especially for acetic acid, propionic acid and butyric acid levels in the intestine. Therefore, it is possible that the intestinal microbiota plays a key role in the intervention of GLE on the alcoholic liver injury in mice exposed to excessive alcohol intake.

### 3.6 GLE modulated intestinal microbiota in mice with excessive alcohol intake

**Fig. 5:**
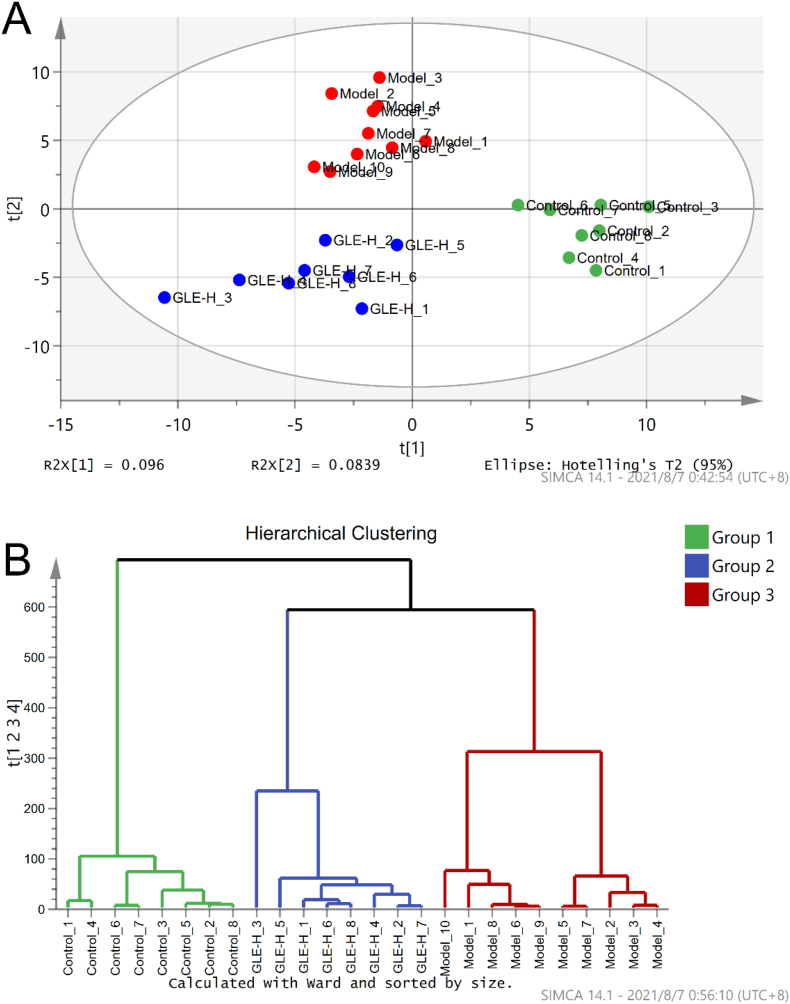
Effects of GLE intervention on intestinal microbial populations in mice with excessive alcohol consumption for consecutive 6 weeks. (A) Score plots of principal component analysis (PC1 × PC2); (B) Hierarchical clustering analysis of intestinal microbiota of different experimental groups drawn based on the relative abundance at genus level.

Excessive drinking is one of the main causes of intestinal microbial disorder, which is closely related to the pathogenesis of alcohol-related liver disease (Caslin et al., 2019). Thus, the intestinal microbial compositions among the experimental groups (the Control, Model and GLE-H groups) were analyze using principal component analysis (PCA) and hierarchical clustering analysis (HCA) (Fig. 5). The result of PCA revealed that the Model group was clearly distinguished from the Control group (Fig. 5A), suggesting the composition of intestinal flora was profoundly affected by excessive alcohol intake, which is agreement with the result of the previous study (Lee and Lee, 2021a). Obviously, GLE intervention significantly changed the intestinal microbial community in mice with excessive alcohol intake. Our previous study showed that GLE intervention altered the composition of intestinal microbiota in mice induced by high-fat diet (Guo et al., 2018). These result of HCA once again proved that oral administration of GLE could influence the intestinal microbiota composition of mice with excessive alcohol intake (Fig. 5B).

**Fig. 6:**
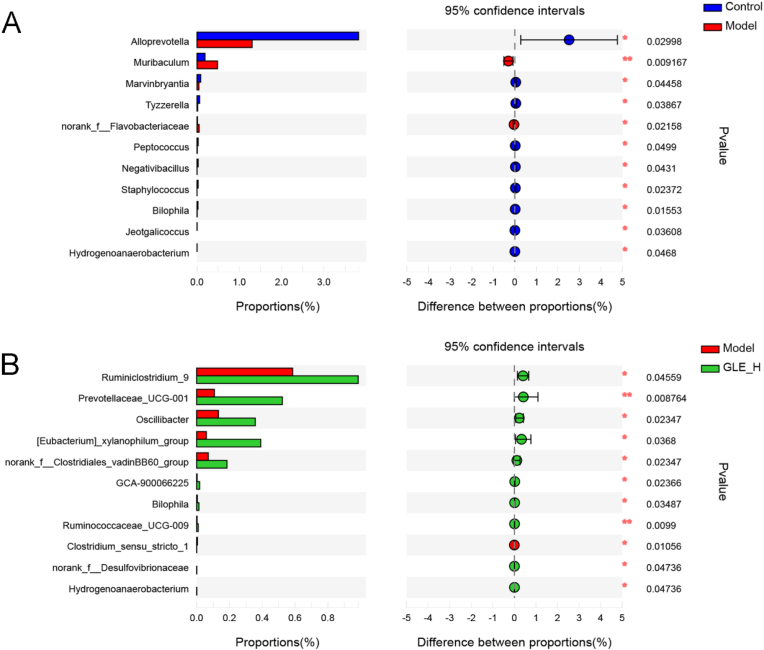
Extended error bar plot comparing the differences in the mean proportions of significantly altered genera and the difference in the proportions of the means. The differences between experimental groups were determined using a Welsh's *t*-test, and the Benjamini-Hochberg procedure was used to control the false-discovery rate due to multiple testing. Corrected P values are shown at right. (A) the Control group *versus* the Model group; (B) the GLE-H group *versus* the Model group.

At the genus level, we found that excessive alcohol consumption profoundly reduced the relative abundance of *Alloprevolla*, *Marvinbryantia*, *Tyzzerella*, *Peptococcus*, *Negativibacillus*, *Staphylococcus*, *Bilophila*, *Jeotgalicoccus* and *Hydrogenoanaerobacterium*, while significantly elevated the proportion of *Muribaculum* and norank_f_Flavobacteriaceae, compared with mice without alcohol treatment (Fig. 6A). It was previously reported that the abundance of *Marvinbryantia* was positively correlated with the level of cecal SCFAs and negatively related to the total antioxidant capacity of liver in LPS-treated mice (Guo et al., 2021). *Tyzzerella* and *Peptococcus* are beneficial to inhibit the development of inflammation and ameliorate the host immunity, partly due to the inhibition of the secretion of lipopolysaccharide, D-lactic acid and TNF-α (Huang et al., 2021b). It was previously reported that *Negativibacillus*, belonging to Ruminococcaceae family, possessed the ability of accelerating the production of butyric acid, which provides energy for intestinal epithelial cells (Huang et al., 2020). In addition, as a gram-positive bacterium, *Jeotgalicoccus* was significantly enriched in the colitis induced by dextran sulfate sodium (Yang et al., 2021). It is thus clear that excessive alcohol intake lead to disorder of intestinal microbial composition, which is consistent with the previous report (Lee and Lee, 2021a). GLE intervention obviously elevated the relative levels of *Ruminiclostridium*_9, *Prevotellaceae*_UCG-001, *Oscillibacter*, [*Eubacterium*]_*xylanophilum*_group, norank_f_*Clostridiates*_*vadinBB60*_group, GCA-900066225, *Bilophila*, *Ruminococcaceae*_UCG-009, norank_f_Desulfovibrionaceae and *Hydrogenoanaerobacterium*, but remarkably reduced the proportion of *Clostridium*_sensu_*stricto*_1 in alcohol-treated mice. *Ruminiclostridium*_9, [Eubacterium]_*xylanophilum*_group and *Ruminococcaceae*_UCG-009 belong to Ruminococcaceae were reported to be the SCFAs producing bacteria, which are helpful to improve the host health by reducing the pH value and inhibiting the growth of intestinal harmful bacteria (Huang, Li, Lee and Chen, 2021a).As one of the most common members of intestinal bacteria, *Prevotellaceae* UCG-001 can produce a variety of enzymes to degrade cellulose and xylan. A previous report also showed that intestinal *Prevotellaceae* UCG-001 could stimulate the AMPK signaling pathway to ameliorate gut health and lipid metabolism (Wang et al., 2021a; Song et al., 2019). The abundance of *Oscillibacter* had been reported to be negatively associated with obesity, fatty liver, inflammation and insulin resistance (Thingholm et al., 2019). As a controversial harmful bacterium, *Desulfovibrionaceae* was previously reported to be closely associated with the production of lipopolysaccharide in intestine (Huang et al., 2021b). It was previously reported that *Clostridium* played an important role in colitis induced by oral administration of dextran sulfate sodium, because *Clostridium* may promote the secretion of inflammatory cytokines (Zhu et al., 2019). *Bilophila* is gram-negative bacterium involved in taurine metabolism, which was previously reported to be benefit for improving lipid metabolism (Ushiroda et al., 2019).

### 3.7 Microbial metabolic function predicted by PICRUSt

Phylogenetic Investigation of Communities by Reconstruction of Unobserved States (PICRUSt) analysis based on 16S rRNA gene sequences and KEGG (Kyoto Encyclopedia of Genes and Genomes) database is widely applied to predict the relationship between phylogeny and metabolism (Smith et al., 2013; Liang et al., 2019). In this study, PICRUSt was used to explore the influence of GLE intervention on the potential metabolic functions of intestinal microbiota in mice with excessive alcohol intake. As shown in Figs. S2 and 12 metabolic functions of intestinal microbiota were significantly changed in mice with excessive alcohol intake. Compared with the Model group, sulfur metabolism [ko00920], butanoate metabolism [ko00650], alpha-linolenic acid metabolism [ko00592], ethylbenzene degradation [ko00642], fatty acid biosynthesis [ko00061], pyruvate metabolism [ko00620] and carbohydrate digestion and absorption [ko04973]) were enriched in mice without alcohol intake (the Control group). On the contrary, african trypanosomiasis [ko05143], PPAR signaling pathway [ko03320], fluorobenzoate degradation [ko00364], bacterial invasion of epithelial cell [ko05100] and bladder cancer [ko05219]) were significantly down-regulated in mice without alcohol intake compared with mice of the Model group (Figs. S2–A). Previous study suggested that long-time excessive alcohol intake is closely associated with the development of liver cancer (Rumgay et al., 2021). After 6 weeks of GLE intervention, cyanoamino acid metabolism [ko00460], phenylpropanoid biosynthesis [ko00940], phosphonate and phosphinate metabolism [ko00440], sulfur metabolism [ko00920], starch and sucrose metabolism [ko00500], pentose and glucuronate interconversions [ko0040] and other glycan degradation [ko00511] were significantly up-regulated, and bacterial secretion system [ko03070], african trypanosomiasis [ko05143], glycerophospholipid metabolism [ko00564], beta-lactam resistance [ko01501] were significantly down-regulated (Figs. S2–B). There were significant differences in microbial metabolic function between the Control group and the GLE-H group, of which galactose metabolism [ko00052], lysosome [ko04212] and other glycan degradation [ko00511]) were significantly enriched, and propanoate metabolism [ko00640], butanoate metabolism [ko00650], nitrotoluene degradation [ko00633], glycerophospholipid metabolism [ko00564], cardiac muscle contraction [ko04260], apoptosis [ko04210], seleno compound metabolism [ko00450] and polycyclic aromatic hydrocarbon degradation [ko00624] were significantly down-regulated by GLE intervention (Figs. S2–C).

### 3.8 Effects of GLE on liver metabolomic profiling in mice with excessive alcohol intake

**Fig. 7:**
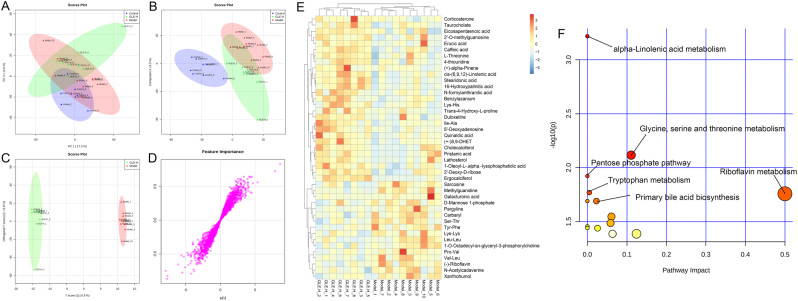
Liver metabolomic profiling by UPLC-QTOF/MS in the positive-ion mode (ESI+). (A) PCA score plot for the Control, Model and GLE-H groups; (B) PLS-DA score plot for the Control, Model and GLE-H groups; (C) OPLS-DA score plot for the GLE-H and Model groups; (D) S-loading plot based on the OPLS-DA analysis model of the GLE-H and Model groups; (E) Heatmap of the relative abundance of significantly different metabolites (VIP value > 1.0, *p* < 0.05) between the Model and GLE-H groups; (F) Metabolic pathway impact prediction based on the KEGG online database. The -ln(*p*) values from the pathway enrichment analysis are indicated on the horizontal axis, and the impact values are indicated on the vertical axis.

**Fig. 8:**
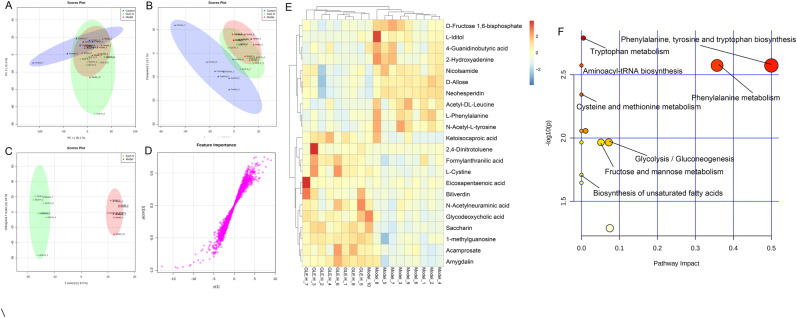
Liver metabolomic profiling by UPLC-QTOF/MS in the negative-ion mode (ESI-). (A) PCA score plot for the Control, Model and GLE-H groups; (B) PLS-DA score plot for the Control, Model and GLE-H groups; (C) OPLS-DA score plot for the GLE-H and Model groups; (D) S-loading plot based on the OPLS-DA analysis model of the GLE-H and Model groups; (E) Heatmap of relative abundance of significantly different metabolites (VIP value > 1.0, *p* < 0.05) between the Model and GLE-H groups; (F) Metabolic pathway impact prediction based on the KEGG online database. The -ln(*p*) values from the pathway enrichment analysis are indicated on the horizontal axis, and the impact values are indicated on the vertical axis.

Metabolomics based on UPLC-QTOF/MS is widely applied to identify and quantify the metabolites in biological organism, which provides quantified information of global changes in metabolic function. In the present study, UPLC-QTOF/MS based liver metabolomics was conducted to further explore the protective mechanism of GLE intervention against alcohol-induced liver injury. To visualize the difference among different experimental groups, multivariate statistical analysis was performed to compare the metabolomic data of liver samples. As shown in Fig. 7A&B and Fig. 8A&B, there was clear difference in the liver metabolic profile among different experimental groups in PCA and PLS-DA score plots, suggesting that excessive alcohol consumption significantly affected liver metabolomic profiling, which is agreement with the previous report (Li et al., 2011). However, it can be seen from the PLS-DA score plots that oral administration of high-dose GLE significantly reversed the damage of liver metabolic function caused by excessive alcohol consumption. Furthermore, OPLS-DA score plots showed significant separation between the Model and GLE-H groups (Fig. 7, Fig. 8C), suggesting GLE intervention could significantly ameliorate the disturbance of liver function in mice exposed to alcohol intake. The S-plots of OPLS-DA exhibited the differences of liver metabonomic profile between the Model and GLE-H groups (VIP >1.0 and *p* < 0.05) (Fig. 7, Fig. 8D).

In the positive-ion (ESI+) mode, a total of 43 potential biomarkers with *p* value < 0.05 and VIP value > 1.0 were successfully identified in the livers between the Model and GLE-H groups (Fig. 7E), of which 27 potential biomarkers (taurocholate, eicosapentaenoic acid, 2′-O-methylguanosine, corticosterone, erucic acid, caffeic acid, L-threonine, 4-thiouridine, (+)-alpha-pinene, cis-(6,9,12)-linolenic acid, stearidonic acid, 16-hydroxypalmitic acid, N-formylanthranilic acid, benzylazanium, Lys-His, trans-4-hydroxy-L-proline, duloxetine, Ile-Ala, 5′-deoxyadenosine, quinaldic acid, (+)-8,9-DHET, cholecalciferol, pristanic acid, lathosterol, 1-oleoyl-L-,alpha-lysophosphatidic acid, 2′-deoxy-D-ribose and ergocalciferol) were significantly up-regulated and 16 potential biomarkers (sarcosine, methylguanidine, galacturonic acid, D-mannose-1-phosphate, pargyline, carbaryl, Ser-Thr, Tyr-Phe, Lys-Lys, Leu-Leu, 1-O-Octadecyl-sn-glyceryl-3-phosphorylcholine, Pro-Val, Val-Leu, (−)-riboflavin, N-acetylcadaverine and xanthohumol) were significantly down-regulated in mice of the GLE-H group, as compared with the Model group. As a conjugated bile acid, taurocholate is mainly secreted in liver cells and participates in the digestion and absorption of fat (Ding et al., 2021). In addition, it has been previously reported that taurocholic acid can exert anti-inflammatory effects by inhibiting the secretion of IL-1β, IFN-γ and TNF-α (Yang et al., 2016). Eicosapentaenoic acid, erucic acid, caffeic acid, cis-(6,9,12)-linolenic acid, stearidonic acid, 16-hydroxypalmitic acid and N-formylanthranilic acid are important products of fatty acid β-oxidation (Bargut et al., 2019), indicating GLE intervention effectively up-regulated the β-oxidation of fatty acid. Ergocalciferol is a type of plant-based vitamin D, which is negatively related to diabetes, hyperlipidemia and other metabolic diseases (Shu et al., 2017). To further visualize the metabolic pathways in response to GLE intervention mice with alcohol intake, metabolic pathway enrichment analysis of liver biomarkers was performed by MetaboAnalyst 4.0 and KEGG. In the ESI + mode, the metabolic pathways significantly altered by high-dose GLE treatment mainly included alpha-linolenic acid metabolism, glycine, serine and threonine metabolism, pentose phosphate pathway, riboflavin metabolism, typtophan metabolism and primary bile acid biosynthesis (Fig. 7F).

In the negative-ion (ESI-) mode, a total of 22 liver differential metabolites were detected between the Model and GLE-H groups, of which 10 metabolites (D-fructose-1,6-bisphosphate, L-iditol, 4-guanidinobutyric acid, 2-hydroxyadenine, nicolsamide, D-allose, neohesperidin, acetyl-DL-leucine, L-phenylalanine and N-acetyl-L-tyrosine) were significantly down-regulated and 47 metabolites were significantly up-regulated in the GLE-H group as compared with the Model group (Fig. 8E). Ketoisocaproic acid, formylanthranilic acid, eicosapentaenoic acid, L-cystine, 2,4-dinitrotoluene, biliverdin, N-acetylneuraminic acid, glycodeoxycholic acid, saccharin, 1-methylguanosine, acamprosate and amygdalin were significantly up-regulated by GLE intervention. Ketoisocaproic acid has been reported to be strongly associated with the blood glucose level, which may be mainly due to the increase in ketoisocaproic acid that can enhance the secretion of insulin (Schulze et al., 2017). Formylanthranilic acid participates in tryptophan metabolism through kynurenine pathway, and plays an important role in maintaining the homeostasis of intestinal mucosa and brain nitrogen (Wang et al., 2021a; Zang et al., 2020). Eicosapentaenoic acid is an omega-3 long-chain polyunsaturated fatty acid, which can effectively regulate the body's oxidative stress level and inhibit the NF-κB signaling pathway (Nikoloff et al., 2021). As a by-product of haem catabolism, biliverdin possesses antioxidant and anti-inflammatory effects by inhibiting the expression of inflammatory cytokines IL-6, MCP-1and TNF-α (Shiels et al., 2020). In addition, N-acetylneuraminic acid has been reported to improve lipid metabolism disorder by reducing serum TC and TG, and reduce oxidative stress injury by elevating the activities of antioxidant enzymes (Guo et al., 2016). Acamprosate is widely used in the treatment of alcohol addiction because it is effective in inhibiting alcohol cravings and relapse (El-Newary et al., 2017). Furthermore, metabolic pathway enrichment of liver biomarkers (ESI-) revealed that phenylalanine, tyrosine and tryptophan biosynthesis, tryptophan metabolism, aminoacyl-tRNA biosynthesis, phenylalanine metabolism, cysteine and methionine metabolism, glycolysis/gluconeogenesis, fructose and mannose metabolism, and biosynthesis of unsaturated fatty acids were enriched to be the main metabolic pathways altered by GLE intervention (Fig. 8F).

### 3.9 Correlations of the key microbial phylotypes with liver metabolites

**Fig. 9:**
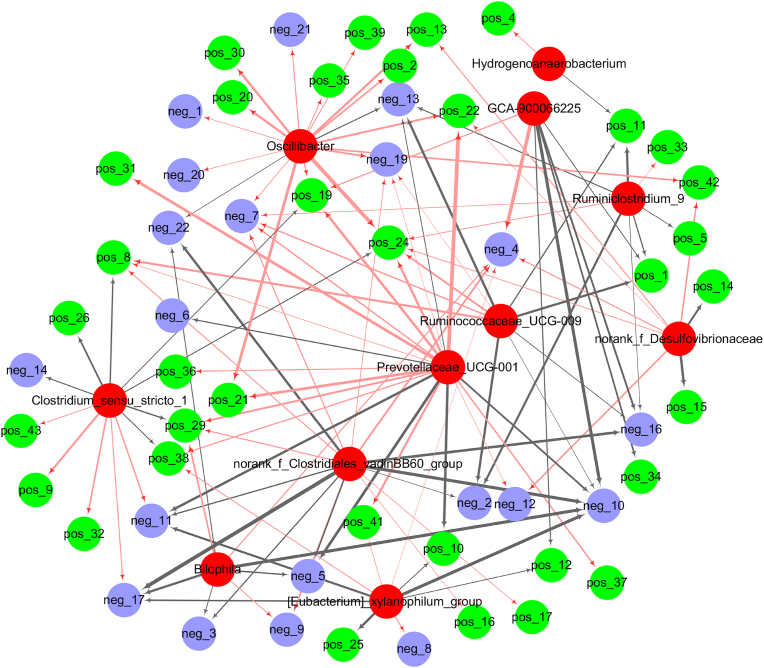
Network visualization according to the Spearman's correlation coefficients between the key intestinal bacterial phylotypes and liver metabolites significantly regulated by GLE intervention. *Red nodes*: the key intestinal microbial phylotypes; *green nodes*: the liver metabolites (ESI+) significantly regulated by high-dose GAA intervention; *blue nodes*: the liver metabolites (ESI-) significantly regulated by high-dose GAA intervention. Red lines and black lines represent positive and negative correlations, respectively. Line width indicates the strength of correlation. Only the significant edges were drawn in the network based on Spearman's correlation (|r| > 0.5, *P* < 0.05). The identification information of liver metabolites in the positive and negative ion modes in the figure was shown in Supplementary Table. S1 and S2, respectively.

Spearman correlation analysis was performed to analyze the association between the key microbial phylotypes with liver metabolites in response to GLE intervention (Fig. 9). Correlation network revealed that *Oscillibacter* was positively correlated with trans-4-hydroxy-l-proline [pos_2], caffeic acid [pos_13], duloxetine [pos_19], 2′-deoxy-d-ribose [pos_20], stearidonic acid [pos_21], lys-his [pos_30], eicosapentaenoic acid [pos_35], erucic acid [pos_39], ketoisocaproic acid [neg_1], acamprosate [neg_7], amygdalin [neg_19], glycodeoxycholic acid [neg_20] and biliverdin [neg_21], but negatively correlated with 2-hydroxyadenine [neg_13] and neohesperidin [neg_22]. *Prevotellaceae*_UCG-001 was positively associated with Ile-Ala [pos_8], duloxetine [pos_19], stearidonic acid [pos_21], cis-(6,9,12)-linolenic acid [pos_22], 16-hydroxypalmitic acid [pos 24], (+-)8,9-DHET [pos_29], pristanic acid [pos_31], lathosterol [pos_37], ergocalciferol [pos_38], acamprosate [neg_7] and L-cystine [neg_12], but negatively associated with Ser-Thr [pos_10], L-phenylalanine [neg_5], acetyl-DL-leucine [neg_6], L-iditol [neg_10], N-acetyl-l-tyrosine [neg_11] and 2-hydroxyadenine [neg_13]. *Clostridium*_sensu_stricto was positively associated with carbaryl [pos_9], Tyr-Phe [pos_32], 1-o-octadecyl-sn-glyceryl-3-phosphorylcholine [pos_43] and n-acetyl-l-tyrosine [neg_11], but negatively associated with Ile-Ala [pos_8], (+)-alpha-pinene [pos_26], 4-thiouridine [pos_29], cholecalciferol [pos_36], ergocalciferol [pos_38] and eicosapentaenoic acid [neg_14]. *Ruminococaceae* UCG-009 was positively correlated with Ile-Ala [pos_8], 16-hydroxypalmitic acid [pos_24] and acamprosate [neg_7], but negatively associated with methylguanidine [pos_1], galacturonic acid [pos_11], caffeic acid [pos 13], 4-guanidinobutyric acid [neg_2], L-iditol [neg 10] and D-fructose 1,6-bisphosphate [neg 16].

### 3.10 Effects of GLE on liver mRNA levels in mice with excessive alcohol intake

**Fig. 10:**
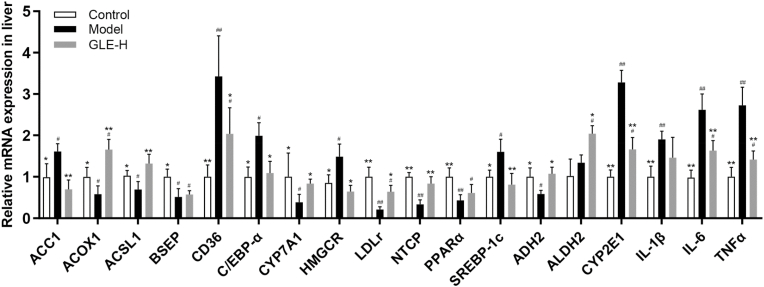
Effects of high-dose GLE administration on the mRNA levels of lipid metabolism and inflammatory response related genes in livers of mice with excessive alcohol consumption for consecutive 6 weeks. ^##^*p* < 0.01 and ^#^*p* < 0.05, versus the Control group; **p < 0.01 and *p < 0.05, versus the Model group.

Liver is an important organ involving in energy metabolism, fatty acid metabolism, bile acid biosynthesis, alcohol metabolism and inflammatory response (Xu et al., 2021). To elaborate the potential mechanism of action by which GLE intervention protects against alcoholic liver injury, transcription levels of the genes related to fatty acid metabolism, bile acid biosynthesis, alcohol metabolism and inflammatory response were further analyzed by RT-qPCR (Fig. 10). Compared with the Control group, the mRNA levels of acetyl-CoA carboxylase 1 (*ACC1*), cluster of differentiation 36 (*CD36*), CCAAT/enhancer binding protein alpha (*C/EBP-α*), hydroxymethylglutaryl-CoA reductase (*HMGCR*) and sterol regulatory element binding transcription factor 1 (*SREBP-1c*) were significantly elevated in mice of the Model group, but the transcription levels of acyl-CoA oxidase 1 (*ACOX1*), acyl-CoA synthetase long-chain family member 1 (*ACSL1*), low density lipoprotein receptor (*LDLr*) and peroxisome proliferator-activated receptor α (*PPARα*) were significantly inhibited (*p* < 0.05). It has been previously reported that *ACC1*, *CD36*, *C/EBP-α* and *SREBP-1c* are involved in the synthesis and accumulation of fatty acids in liver (Wang et al., 2021b). *SREBP-1c* is an important regulator controlling the transcriptional expression of *ACC1* and *CD36* genes, which act as the rate limiting-enzymes for de novo fatty acid synthesis and fatty acid absorption, respectively (Guo et al., 2020b). *C/EBP-α* is an important transcription factor regulating the adipocyte differentiation in T3T-L1 cell (Balakrishnan et al., 2018). Therefore, inhibiting the expression of liver *ACC1*, *CD36*, *C/EBP-α* and *SREBP-1c* is beneficial to prevent the excessive accumulation of liver fatty acids. *ASCL1* is involved in the shift of *PPARα* activation, and it well known that *PPARα* is a mainly modulator in the β-oxidation of fatty acids by regulating the mRNA expression of *ACOX1* (a lipid-oxidation enzyme) (Huang et al., 2020; Lee et al., 2021b). The main function of *ACSL1* is to promote the transfer of fatty acids as well as the synthesis of triacylglycerol, and determine the intake of exogenous fatty acids from adipose tissue (Zhao et al., 2020). It has been widely recognized that *PPARα* is the main regulator controlling the β-oxidation of fatty acids, which can regulate the liver lipid homeostasis by regulating the expression of *ACOX1* (an enzyme involved in lipid-oxidation) (Jia et al., 2019). Therefore, the activation of *ACOX1*, *ACSL1* and *PPARα* transcription may accelerate the oxidation of fatty acids, which would further ameliorate liver injury induced by excessive alcohol intake. Our result indicated that GLE intervention protected against the pathological process of alcoholic liver injury partly by inhibiting fatty acid synthesis and accelerating fatty acid oxidation.

In addition, the mRNA expressions of key genes including cholesterol-7α-hydroxylase (*CYP7A1*), bile salt export pump (*BSEP*) and Na+/taurocholate cotransporting polypeptide (*NTCP*) related to bile acid biosynthesis and metabolism were analyzed (Duan et al., 2021). Compared with the Control group, the transcription levels of *CYP7A1*, *BSEP* and *NTCP* were obviously down-regulated in mice with excessive alcohol intake (the Model group) (*p* < 0.05). After 6 weeks of GLE intervention, the mRNA expressions of *CYP7A1* and *NTCP* were significantly up-regulated (*p* < 0.05). It is clear that the conversion of liver cholesterol into bile acids is conducive to the prevention of some diseases related to cholesterol metabolism disorders (Lv et al., 2019). *CYP7A1* is generally considered to be the first rate-limiting enzyme for liver cholesterol conversion, thus *CYP7A1* activation promotes the bile acids biosynthesis from cholesterol in the liver (Lv et al., 2019). As a key transporter involving in the excretion of bile acids, *NCTP* is responsible for the excretion of bile acids from the liver (König et al., 2014). Therefore, activating *NTCP* is beneficial for relieving cholesterol metabolism disorder by promoting the excretion of bile acids from the liver to the intestine, which is helpful to avoid the liver damage induced by excessive accumulation of bile acids.

It has been widely accepted that the up-regulation of alcohol metabolism is helpful to reduce the development of alcoholic liver injury (Liu et al., 2020). In addition to improving cholesterol metabolism, high-dose GAA intervention also protected against alcoholic liver injury by regulating the mRNA transcription level of genes related to alcohol metabolism. In detail, high-dose GLE intervention (100 mg/kg b.w.) significantly down-regulated the mRNA levels of cytochrome P4502E1 (*CYP2E1*), but significantly up-regulated the mRNA expression of alcohol dehydrogenase 2 (*ADH2*) and aldehyde dehydrogenase 2 (*ALDH2*) in mice with excessive alcohol intake (*p* < 0.05). *CYP2E1* is considered to the vital pathway of toxicity in hepatic and extra-hepatic cells induced by alcohol and acetaminophen through regulating the host oxidative stress (Kouam et al., 2020). Previous investigation had showed that *CYP2E1* activity was abnormally elevated in patients with alcoholic liver injury, because long-term alcohol consumption can aggravate oxidative stress and the formation of toxic metabolites by stimulating *CYP2E1* activity (Rahman et al., 2019). *ADH2* is a key enzyme that converts alcohol into acetaldehyde, and it participates in the first-step of ethanol metabolism in human body (Wang et al., 2020). Acetaldehyde can be further metabolized to acetic acid with relatively low toxicity by activating *ALDH2* activity (Wang et al., 2020). Thus, the down-regulation of *CYP2E1* and the up-regulation of *ALD2* and *ALDH2* are beneficial for preventing the pathological process of alcoholic liver injury.

Inflammation is generally considered to be an important biomarker of the degree of alcoholic liver damage (Chang et al., 2013). Our results showed that the mRNA expressions of *IL-1β*, *IL-6* and *TNF-α* were significantly increased in mice with excessive alcohol intake. *TNF-α* plays an important role in some inflammatory diseases by up-regulating the NF-κB signaling pathway and accelerating the release of *IL-6* and *IL-1β* (Dendoncker et al., 2019). Previous study suggested alcohol treatment significantly increased the serum levels of *IL-1β*, *IL-6* and *TNF-α*, which is consistent with our findings (Nivukoski et al., 2021). Interestingly, high-dose GLE intervention can significantly reverse the abnormal transcription levels of IL-6 and *TNF-α* in liver induced by excessive alcohol intake (*p* < 0.01).

## 4 Conclusion

In this study, the protective effects of GLE on alcohol-induced liver injury and its possible mechanism of action were explored. Phytochemical analysis based on HPLC-QTOF/MS revealed that GLE is rich in ganoderic acids. Oral administration of GLE obviously ameliorated alcoholic liver injury and intestinal microbial disturbance in mice exposed to alcohol consumption. The potential protective mechanisms of GLE intervention against the pathological process of alcoholic liver injury were elucidated through intestinal microbiomics, liver metabolomics and RT-qPCR. These findings preliminarily suggest that dietary supplementation of GLE ameliorates alcoholic liver injury possibly by modulating the composition of intestinal microbiota and liver metabonomic profile, and regulating the mRNA levels of key genes related to fatty acid metabolism, bile acid biosynthesis and inflammatory response. This study reveals that ganoderic acid has potential beneficial effects in preventing alcohol-induced liver injury, and is expected to become a promising functional food ingredient. In further study, the protective mechanisms of ganoderic acid against alcoholic liver injury and oxidative stress need to be clarified through transcriptome and proteomics, as well as clinical crowd trials combined with multi-omics technology, so as to provide more credible references for the development of a promising functional food to improve or prevent alcoholic liver injury.

## CRediT authorship contribution statement

**Wei-Ling Guo:** Project administration, Conceptualization, Investigation, Writing – original draft. **Ying-Jia Cao:** Conceptualization, Investigation, Writing – original draft. **Shi-Ze You:** Conceptualization, Investigation, Writing – original draft. **Qi Wu:** Visualization, Writing – review & editing. **Fang Zhang:** Methodology, Software. **Jin-Zhi Han:** Methodology, Software. **Xu-Cong Lv:** Resources, Supervision, Writing – review & editing, Conceptualization, Funding acquisition. **Ping-Fan Rao:** Supervision, Writing – review & editing. **Lian-Zhong Ai:** Supervision, Writing – review & editing. **Li Ni:** Resources, Writing – review & editing, Validation.

## Declaration of competing interest

The authors declare that they have no known competing financial interests or personal relationships that could have appeared to influence the work reported in this paper.
