# Corrigendum to “Ganoderic acids-rich ethanol extract from Ganoderma lucidum protects against alcoholic liver injury and modulates intestinal microbiota in mice with excessive alcohol intake” [Curr. Res. Food Sci. 5(2022) 515–530]

**DOI:** 10.1016/j.crfs.2022.07.001

**Published:** 2022-07-14

**Authors:** Wei-Ling Guo, Ying-Jia Cao, Shi-Ze You, Qi Wu, Fang Zhang, Jin-Zhi Han, Xu-Cong Lv, Ping-Fan Rao, Lian-Zhong Ai, Li Ni

**Affiliations:** a Institute of Food Science and Technology, College of Biological Science and Technology, Fuzhou University, Fuzhou, Fujian, 350108, China; b School of Clinical Medicine, Fujian Medical University, Fuzhou, Fujian, 350122, China; c National Engineering Research Center of JUNCAO Technology, Fujian Agriculture and Forestry University, Fuzhou, Fujian, 350002, China; d School of Medical Instruments and Food Engineering, University of Shanghai for Science and Technology, Shanghai, 200093, China; e School of Food Science and Technology, Jiangnan University, Wuxi, Jiangsu, 214122, China

The authors regret to inform that during the final submission of the figures in this manuscript's publication, the liver histopathological image of the Silymarin 100 group detected by hematoxylin-eosin (H&E) staining in Fig. 3(B) was duplicated by mistake when we laid out the images. The image for the Silymarin 100 group in Fig. 3(B) was inadvertently replaced with the image of another group carried out in the same batch. Nevertheless, we confirm that this mistake did not influence the research results and scientific conclusions of the article in any way.

In the corrigendum Figure, we have provided a correct image for the Silymarin 100 group in Fig. 3(B). The only change in the panel of Fig. 3(B) is the Silymarin 100 group, the rest of the figure is identical to the published version. The authors would like to apologize for this oversight and for any confusion that it has caused.

The incorrect version for Fig. 3(B):

**Figure:**
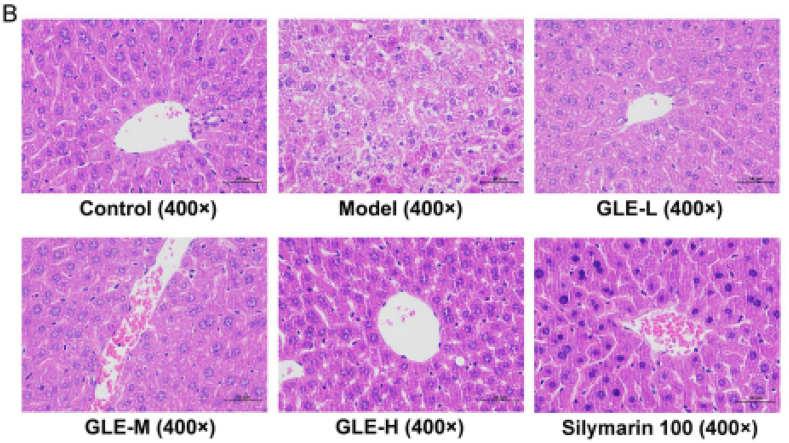

Fig. 3. Effects of GLE intervention on the liver histopathological features in mice with excessive alcohol consumption.

The corrected version for Fig. 3(B):

**Figure:**
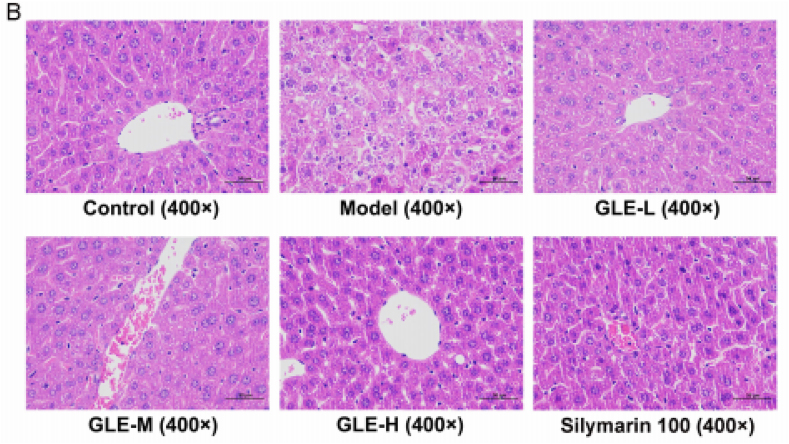

Fig. 3. Effects of GLE intervention on the liver histopathological features in mice with excessive alcohol consumption.

