# Supplementary material for: Ganoderic acids-rich ethanol extract from Ganoderma lucidum protects against alcoholic liver injury and modulates intestinal microbiota in mice with excessive alcohol intake
Source: Curr Res Food Sci. 2022 Feb 24;5:515–30. doi: 10.1016/j.crfs.2022.02.013 (PMC8913248; doi:10.1016/j.crfs.2022.02.013)
Supplement: Multimedia component 1 [file mmc1.doc]

**Supplementary Materials**

**Fig. S1.** Phytochemical analysis of *G. lucidum* ethanol extract (GLE) performed through high performance liquid chromatography (HPLC) coupled with a QTOF electrospray ionization MS system.


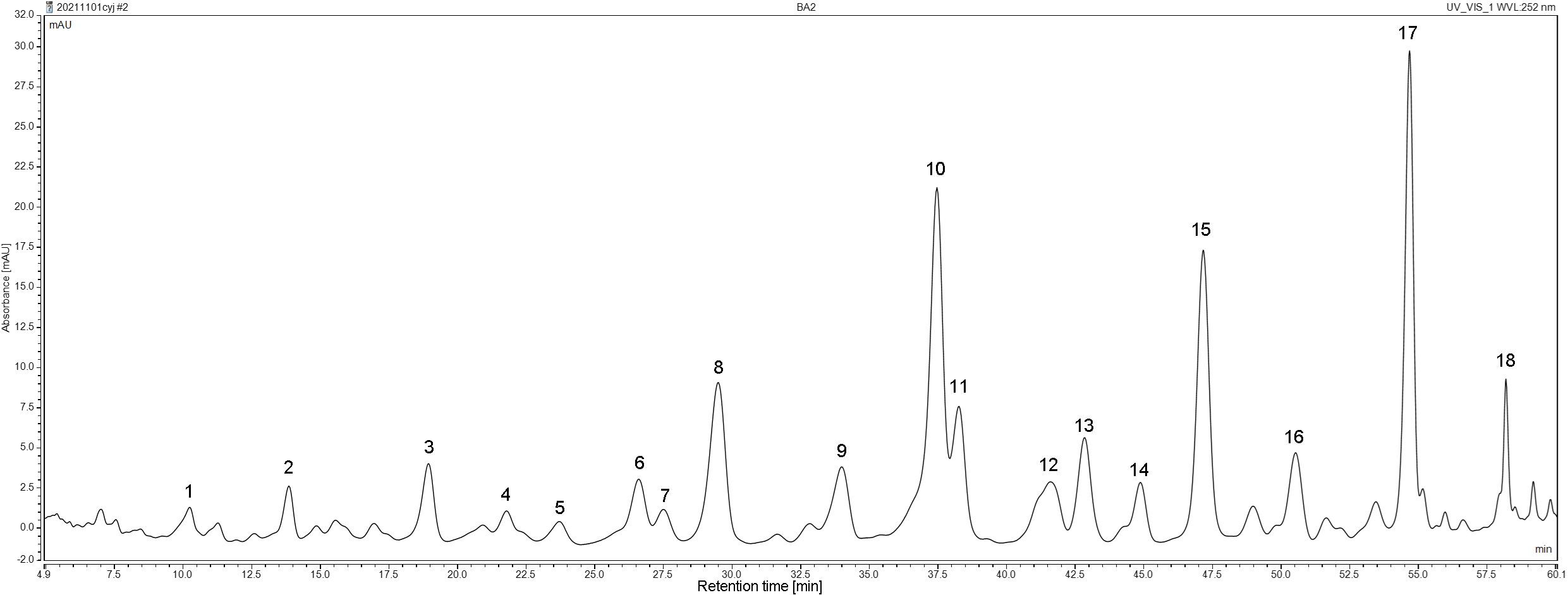


**Instrumentation, chromatography and ESI-MS operating conditions:**

Agilent 1260 HPLC system (Agilent, CA, USA) equipped with a diode-array detector were used to the purity verification of ganoderic acid A (GAA) in this study. Waters Acquity BEH C18 column (2.1 × 150 mm i.d., 1.7 μm, Waters, Milford, MA, USA). was used for separation. The [mobile phase](https://www.sciencedirect.com/topics/chemistry/mobile-phase-composition) was consisted of 0.2% acetic acid in water (A) and acetonitrile (B) with [gradient elution](https://www.sciencedirect.com/topics/chemistry/gradient-elution): 0–25 min, 28%B–30%B; 25–50 min, 30%B–39%B; 50–60 min, 39%B–60%B; 60–70 min, 60%B–100%B. The flow rate was 0.3 mL/min, injection volume was 1 μL and the column temperature was 25 °C, and the detection wavelength was set at 257 nm. A high-resolution QTOF [mass spectrometer](https://www.sciencedirect.com/topics/physics-and-astronomy/mass-spectrometers) (Agilent 6530, Agilent, CA, USA) equipped with an [electrospray ionization](https://www.sciencedirect.com/topics/pharmacology-toxicology-and-pharmaceutical-science/electrospray-ionization) (ESI) source was operated in the [negative ion](https://www.sciencedirect.com/topics/pharmacology-toxicology-and-pharmaceutical-science/anions) mode. The mass range was set at m/z 100–1500 in the full scan mode. The capillary voltage was set at 3500 V. The source temperature was set at 250 °C. Nitrogen was used as the drying gas. The gas flow rate was set at 8 L/min. MS2 data analysis of the three highest intensive ion fragments was intelligently performed in real time.

**Fig. S2.** The functional prediction analysis of intestinal microbial population among the Control, Model and GLE-H groups based on PICRUSt. (A) the Control group versus the Model group; (B) the GLE-H group versus the Model group; (C) the GLE-H group versus the Control group. p < 0.05 was regarded as statistically signifcant.


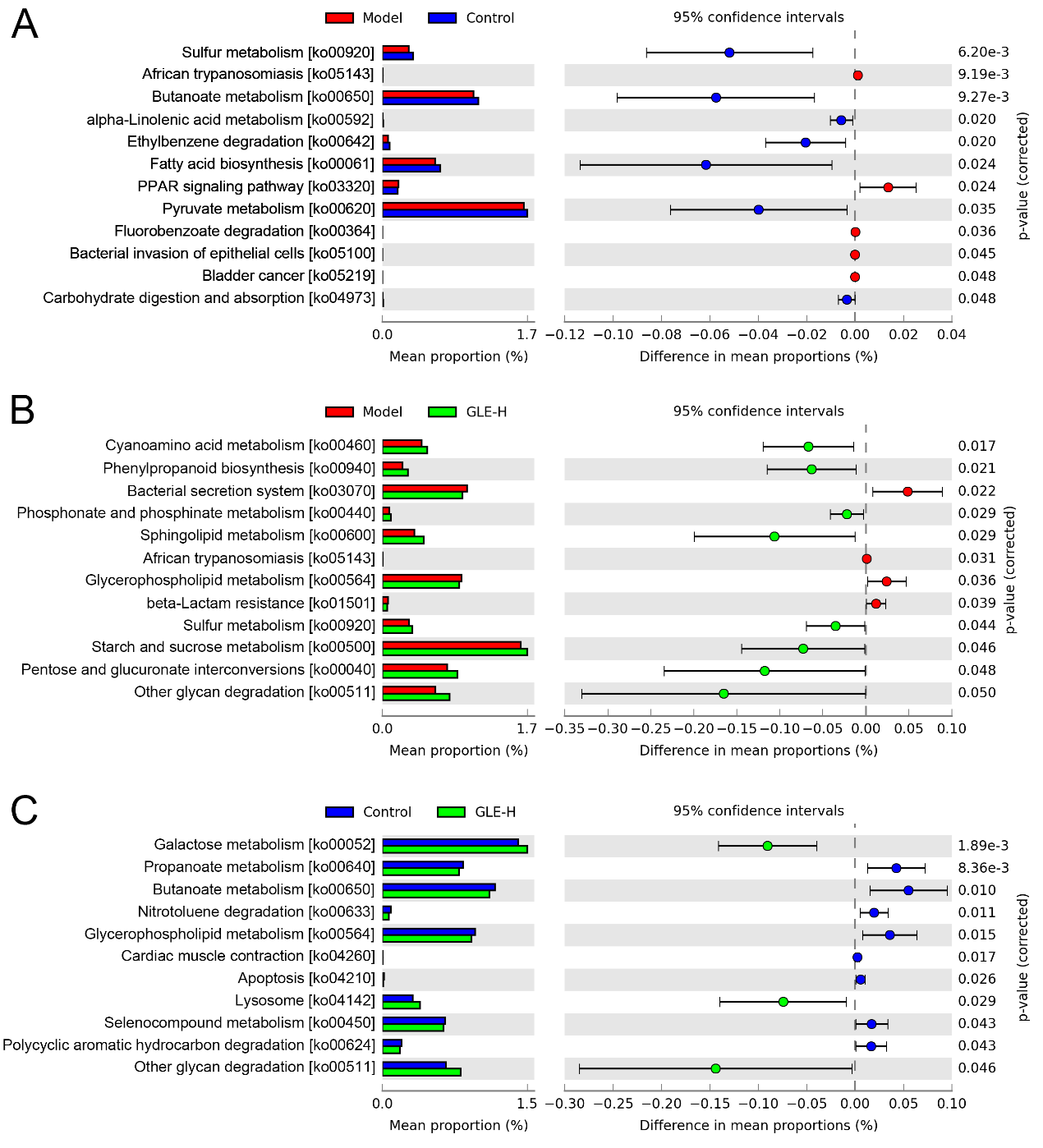


**Table. S1.** The relative abundance of significantly different metabolites between the Model and GLE-H groups by UPLC-QTOF/MS in ESI+ mode.

| **ID.** | **Metabolite name** | **Model_1** | **Model_2** | **Model_3** | **Model_4** | **Model_5** | **Model_6** | **Model_7** | **Model_8** | **Model_9** | **Model_10** | **GLE-H_1** | **GLE-H_2** | **GLE-H_3** | **GLE-H_4** | **GLE-H_5** | **GLE-H_6** | **GLE-H_7** | **GLE-H_8** |
| --- | --- | --- | --- | --- | --- | --- | --- | --- | --- | --- | --- | --- | --- | --- | --- | --- | --- | --- | --- |
| **pos_1** | **Methylguanidine** | 0.0332 | 0.0552 | 0.0463 | 0.0727 | 0.1231 | 0.0424 | 0.0933 | 0.0882 | 0.0382 | 0.0690 | 0.0398 | 0.0409 | 0.0371 | 0.0441 | 0.0289 | 0.0448 | 0.0191 | 0.0463 |
| **pos_2** | **Trans-4-Hydroxy-L-proline** | 0.0349 | 0.0314 | 0.0389 | 0.0298 | 0.0141 | 0.0142 | 0.0487 | 0.1109 | 0.0416 | 0.0381 | 0.1440 | 0.0303 | 0.0927 | 0.0976 | 0.0402 | 0.0344 | 0.1467 | 0.0613 |
| **pos_3** | **L-Threonine** | 0.7676 | 1.2437 | 2.1319 | 1.5772 | 1.0321 | 1.2698 | 1.2330 | 1.2365 | 1.5447 | 1.3867 | 1.8603 | 1.3868 | 1.5216 | 1.5270 | 1.6370 | 1.8313 | 1.7738 | 1.6452 |
| **pos_4** | **N-formylanthranilic acid** | 0.0391 | 0.0364 | 0.0555 | 0.0581 | 0.0554 | 0.0403 | 0.0561 | 0.0755 | 0.0701 | 0.0363 | 0.0445 | 0.0936 | 0.0559 | 0.1082 | 0.0496 | 0.0981 | 0.0871 | 0.0868 |
| **pos_5** | **Sarcosine** | 0.0540 | 0.0771 | 0.0864 | 0.1354 | 0.0993 | 0.0800 | 0.0864 | 0.1020 | 0.0656 | 0.0749 | 0.0842 | 0.0504 | 0.0478 | 0.1013 | 0.0497 | 0.0748 | 0.0383 | 0.0730 |
| **pos_6** | **Quinaldic acid** | 0.0304 | 0.0457 | 0.0616 | 0.0486 | 0.0569 | 0.0611 | 0.0504 | 0.0767 | 0.0458 | 0.0758 | 0.0635 | 0.1440 | 0.0589 | 0.0657 | 0.0808 | 0.1077 | 0.0749 | 0.0709 |
| **pos_7** | **Pargyline** | 0.0399 | 0.0646 | 0.0728 | 0.0463 | 0.0368 | 0.0323 | 0.0252 | 0.0806 | 0.1353 | 0.0784 | 0.0412 | 0.0246 | 0.0449 | 0.0453 | 0.0275 | 0.0274 | 0.0349 | 0.0380 |
| **pos_8** | **Ile-Ala** | 0.0540 | 0.0838 | 0.0984 | 0.0666 | 0.0885 | 0.1116 | 0.0794 | 0.1054 | 0.1055 | 0.0899 | 0.1695 | 0.2067 | 0.0733 | 0.1531 | 0.1181 | 0.1227 | 0.1323 | 0.1126 |
| **pos_9** | **Carbaryl** | 0.6773 | 0.4902 | 0.6617 | 0.5190 | 0.4310 | 0.5255 | 0.4400 | 0.6023 | 0.6093 | 0.6562 | 0.4365 | 0.4567 | 0.5268 | 0.5001 | 0.4260 | 0.4905 | 0.3273 | 0.4097 |
| **pos_10** | **Ser-Thr** | 0.0481 | 0.0426 | 0.0509 | 0.0253 | 0.0204 | 0.0232 | 0.0362 | 0.0395 | 0.0376 | 0.0377 | 0.0156 | 0.0098 | 0.0195 | 0.0315 | 0.0236 | 0.0351 | 0.0196 | 0.0283 |
| **pos_11** | **Galacturonic acid** | 0.0091 | 0.0111 | 0.0159 | 0.0525 | 0.1613 | 0.0331 | 0.0098 | 0.0132 | 0.0064 | 0.0116 | 0.0286 | 0.0072 | 0.0100 | 0.0078 | 0.0042 | 0.0083 | 0.0020 | 0.0068 |
| **pos_12** | **Pro-Val** | 0.0658 | 0.0659 | 0.0918 | 0.0980 | 0.0698 | 0.0742 | 0.1026 | 0.1947 | 0.0955 | 0.0964 | 0.0543 | 0.0495 | 0.0471 | 0.0775 | 0.0769 | 0.0579 | 0.0907 | 0.0865 |
| **pos_13** | **Caffeic acid** | 0.0071 | 0.0273 | 0.0404 | 0.0120 | 0.0179 | 0.0126 | 0.0150 | 0.0222 | 0.0307 | 0.0411 | 0.0301 | 0.0274 | 0.0346 | 0.0324 | 0.0277 | 0.0495 | 0.0444 | 0.0430 |
| **pos_14** | **Val-Leu** | 0.2188 | 0.1771 | 0.2976 | 0.2309 | 0.1976 | 0.1844 | 0.3367 | 0.2965 | 0.2068 | 0.2079 | 0.2161 | 0.1810 | 0.1402 | 0.1885 | 0.1933 | 0.1674 | 0.1990 | 0.1800 |
| **pos_15** | **Leu-Leu** | 0.6399 | 0.5460 | 0.9025 | 0.7494 | 0.9543 | 0.7973 | 0.5920 | 1.0122 | 0.9160 | 1.2244 | 0.9045 | 0.6538 | 0.5326 | 0.7603 | 0.6303 | 0.4947 | 0.5975 | 0.4998 |
| **pos_16** | **5'-Deoxyadenosine** | 0.4202 | 0.3998 | 0.5192 | 0.3336 | 0.4132 | 0.3974 | 0.4270 | 0.4167 | 0.3892 | 0.4411 | 0.6407 | 0.6255 | 0.4041 | 0.5848 | 0.5252 | 0.4962 | 0.4428 | 0.4257 |
| **pos_17** | **Benzylazanium** | 0.2953 | 0.2739 | 0.3396 | 0.2645 | 0.3532 | 0.2481 | 0.2086 | 0.2833 | 0.3977 | 0.3438 | 0.4546 | 0.3244 | 0.2715 | 0.4277 | 0.2972 | 0.4993 | 0.4599 | 0.3156 |
| **pos_18** | **N-Acetylcadaverine** | 0.3672 | 0.4630 | 0.4595 | 0.3108 | 0.2725 | 0.4349 | 0.3252 | 0.3520 | 0.4912 | 0.2112 | 0.2735 | 0.3008 | 0.2789 | 0.3706 | 0.1503 | 0.2727 | 0.2564 | 0.3262 |
| **pos_19** | **Duloxetine** | 0.1120 | 0.0997 | 0.1553 | 0.1021 | 0.1222 | 0.1409 | 0.1564 | 0.3371 | 0.1584 | 0.0510 | 0.2533 | 0.2395 | 0.2148 | 0.1777 | 0.1356 | 0.2006 | 0.1801 | 0.2083 |
| **pos_20** | **2'-Deoxy-D-ribose** | 0.3671 | 0.3522 | 0.4772 | 0.4198 | 0.6827 | 0.6370 | 0.5298 | 0.4443 | 0.7715 | 0.7217 | 0.7062 | 0.6106 | 0.7011 | 0.8348 | 0.7013 | 0.6621 | 0.8243 | 0.6392 |
| **pos_21** | **Stearidonic acid** | 0.2098 | 0.3082 | 0.3859 | 0.2743 | 0.4064 | 0.3405 | 0.4593 | 0.3849 | 0.5798 | 0.2916 | 0.5715 | 0.4488 | 0.7975 | 0.4891 | 0.6421 | 0.6790 | 0.7680 | 0.4671 |
| **pos_22** | **cis-(6,9,12)-Linolenic acid** | 0.3312 | 0.4350 | 0.5125 | 0.4270 | 0.6814 | 0.5164 | 0.6084 | 0.5369 | 0.6159 | 0.4911 | 0.6420 | 0.5797 | 0.7463 | 0.5830 | 0.6228 | 0.8871 | 0.9047 | 0.6337 |
| **pos_23** | **2'-O-methylguanosine** | 0.4712 | 0.7847 | 1.3633 | 0.4874 | 0.6272 | 0.6857 | 0.3911 | 0.7907 | 0.7511 | 1.9093 | 1.3505 | 1.5168 | 1.1214 | 0.8698 | 1.4993 | 1.1808 | 0.8783 | 0.8553 |
| **pos_24** | **16-Hydroxypalmitic acid** | 0.2868 | 0.4193 | 0.4359 | 0.3779 | 0.3612 | 0.4656 | 0.4818 | 0.4864 | 0.6166 | 0.3486 | 0.5855 | 0.4674 | 0.8811 | 0.5779 | 0.6662 | 0.8401 | 0.9051 | 0.6235 |
| **pos_25** | **D-Mannose 1-phosphate** | 0.1308 | 0.1428 | 0.1539 | 0.0991 | 0.1075 | 0.0882 | 0.0604 | 0.0615 | 0.1493 | 0.0490 | 0.0369 | 0.0204 | 0.0514 | 0.0730 | 0.0790 | 0.1647 | 0.0674 | 0.0410 |
| **pos_26** | **(+)-alpha-Pinene** | 0.0758 | 0.0851 | 0.1113 | 0.1145 | 0.0414 | 0.0830 | 0.1482 | 0.1108 | 0.1003 | 0.0432 | 0.1215 | 0.1519 | 0.0809 | 0.1255 | 0.1226 | 0.2229 | 0.3454 | 0.0783 |
| **pos_27** | **Lys-Lys** | 0.1358 | 0.0885 | 0.0654 | 0.0762 | 0.0976 | 0.0882 | 0.1370 | 0.1887 | 0.0687 | 0.2235 | 0.0418 | 0.0775 | 0.0208 | 0.1460 | 0.0291 | 0.0947 | 0.0695 | 0.0911 |
| **pos_28** | **4-thiouridine** | 0.0248 | 0.0819 | 0.1188 | 0.0634 | 0.0454 | 0.0535 | 0.0800 | 0.0316 | 0.0667 | 0.0648 | 0.0555 | 0.0591 | 0.0886 | 0.0955 | 0.0892 | 0.1188 | 0.1066 | 0.0940 |
| **pos_29** | **(+-)8,9-DHET** | 0.1389 | 0.1876 | 0.2415 | 0.1546 | 0.2562 | 0.2481 | 0.2165 | 0.2580 | 0.2415 | 0.2765 | 0.3696 | 0.3707 | 0.2544 | 0.2783 | 0.2286 | 0.4982 | 0.3192 | 0.2668 |
| **pos_30** | **Lys-His** | 0.2696 | 0.4677 | 0.5544 | 0.4373 | 0.2565 | 0.3468 | 0.2924 | 0.4320 | 0.6433 | 0.4740 | 0.6317 | 0.3278 | 0.4861 | 0.9251 | 0.7102 | 0.8714 | 0.6541 | 0.4808 |
| **pos_31** | **Pristanic acid** | 0.0515 | 0.0868 | 0.1592 | 0.0777 | 0.3917 | 0.4590 | 0.1033 | 0.0548 | 0.1210 | 0.2313 | 0.3324 | 0.3298 | 0.1696 | 0.1752 | 0.1999 | 0.2967 | 0.4118 | 0.2382 |
| **pos_32** | **Tyr-Phe** | 0.1406 | 0.0385 | 0.0713 | 0.0895 | 0.0494 | 0.1136 | 0.0718 | 0.0463 | 0.0855 | 0.1027 | 0.0508 | 0.0318 | 0.0503 | 0.0394 | 0.0562 | 0.0366 | 0.0610 | 0.0772 |
| **pos_33** | **Corticosterone** | 0.3590 | 0.2776 | 0.2277 | 0.3592 | 0.3077 | 0.3659 | 0.4096 | 0.3464 | 0.1249 | 0.2933 | 0.3135 | 0.5428 | 0.3534 | 0.3921 | 0.4360 | 0.4065 | 0.3017 | 0.9034 |
| **pos_34** | **(-)-Riboflavin** | 0.5176 | 0.6927 | 0.6809 | 0.3798 | 0.4970 | 0.6370 | 0.8535 | 0.7215 | 0.5625 | 0.4681 | 0.3841 | 0.4788 | 0.4311 | 0.4445 | 0.4833 | 0.3282 | 0.4621 | 0.6079 |
| **pos_35** | **Eicosapentaenoic acid** | 0.1507 | 0.0271 | 0.0224 | 0.1405 | 0.0156 | 0.0169 | 0.2032 | 0.2196 | 0.2459 | 0.3101 | 0.1993 | 0.2394 | 0.2178 | 0.2440 | 0.1689 | 0.2818 | 0.1977 | 0.2318 |
| **pos_36** | **Cholecalciferol** | 0.0238 | 0.0360 | 0.0916 | 0.0533 | 0.2751 | 0.2117 | 0.0610 | 0.0194 | 0.0510 | 0.2888 | 0.2287 | 0.3427 | 0.1535 | 0.1450 | 0.0951 | 0.1930 | 0.1763 | 0.1753 |
| **pos_37** | **Lathosterol** | 0.0676 | 0.0968 | 0.2359 | 0.1015 | 0.5882 | 0.3090 | 0.0981 | 0.0432 | 0.0803 | 0.2415 | 0.3421 | 0.3483 | 0.1701 | 0.2309 | 0.1425 | 0.4370 | 0.3989 | 0.2394 |
| **pos_38** | **Ergocalciferol** | 0.0147 | 0.0160 | 0.0154 | 0.0130 | 0.0841 | 0.0137 | 0.0154 | 0.0138 | 0.0152 | 0.0198 | 0.1532 | 0.0399 | 0.1191 | 0.1347 | 0.1566 | 0.0954 | 0.0879 | 0.1334 |
| **pos_39** | **Erucic acid** | 0.0439 | 0.0492 | 0.0417 | 0.0747 | 0.0541 | 0.0482 | 0.0596 | 0.0791 | 0.0629 | 0.2061 | 0.1435 | 0.0703 | 0.0952 | 0.0755 | 0.0928 | 0.1040 | 0.0920 | 0.1197 |
| **pos_40** | **Xanthohumol** | 0.0314 | 0.0459 | 0.0668 | 0.0354 | 0.0520 | 0.0544 | 0.0517 | 0.0460 | 0.0584 | 0.0361 | 0.0352 | 0.0267 | 0.0426 | 0.0409 | 0.0301 | 0.0367 | 0.0436 | 0.0454 |
| **pos_41** | **1-Oleoyl-L-.alpha.-lysophosphatidic acid** | 3.4110 | 2.8284 | 4.6168 | 2.3528 | 4.6629 | 3.4602 | 4.4373 | 3.0441 | 3.3876 | 4.5919 | 6.6406 | 5.1483 | 5.3682 | 4.3996 | 4.2310 | 4.6929 | 4.3775 | 3.7952 |
| **pos_42** | **Taurocholate** | 0.3008 | 0.3381 | 0.4239 | 0.4031 | 0.3588 | 0.3526 | 0.3261 | 0.4815 | 0.4169 | 0.4227 | 0.4675 | 0.3844 | 0.4434 | 0.3702 | 0.4421 | 0.4954 | 0.4611 | 0.5876 |
| **pos_43** | **1-O-Octadecyl-sn-glyceryl-3-phosphorylcholine** | 0.1590 | 0.1231 | 0.1870 | 0.1454 | 0.1897 | 0.2125 | 0.1330 | 0.1776 | 0.1819 | 0.2502 | 0.1694 | 0.1009 | 0.1026 | 0.1109 | 0.1320 | 0.1537 | 0.0912 | 0.1896 |

* Data were calibrated by the peak area of the internal standard.

**Table. S2.** The relative abundance of significantly different metabolites between the Model and GLE-H groups by UPLC-QTOF/MS in ESI- mode.

| **ID.** | **Metabolite name** | **GLE-H_1** | **GLE-H_2** | **GLE-H_3** | **GLE-H_4** | **GLE-H_5** | **GLE-H_6** | **GLE-H_7** | **GLE-H_8** | **Model_1** | **Model_2** | **Model_3** | **Model_4** | **Model_5** | **Model_6** | **Model_7** | **Model_8** | **Model_9** | **Model_10** |
| --- | --- | --- | --- | --- | --- | --- | --- | --- | --- | --- | --- | --- | --- | --- | --- | --- | --- | --- | --- |
| **neg_1** | **Ketoisocaproic acid** | 0.0977 | 0.1066 | 0.0961 | 0.1312 | 0.0454 | 0.0508 | 0.0547 | 0.0684 | 0.0244 | 0.0231 | 0.0301 | 0.0182 | 0.0352 | 0.0204 | 0.0215 | 0.1296 | 0.0735 | 0.0582 |
| **neg_2** | **4-Guanidinobutyric acid** | 0.0006 | 0.0006 | 0.0009 | 0.0011 | 0.0005 | 0.0005 | 0.0005 | 0.0006 | 0.0005 | 0.0011 | 0.0005 | 0.0011 | 0.0013 | 0.0007 | 0.0015 | 0.0016 | 0.0010 | 0.0012 |
| **neg_3** | **D-Allose** | 0.0048 | 0.0006 | 0.0051 | 0.0036 | 0.0047 | 0.0042 | 0.0070 | 0.0057 | 0.0078 | 0.0095 | 0.0071 | 0.0086 | 0.0074 | 0.0071 | 0.0071 | 0.0060 | 0.0078 | 0.0027 |
| **neg_4** | **Formylanthranilic acid** | 0.0465 | 0.0492 | 0.0727 | 0.0524 | 0.0432 | 0.0665 | 0.0386 | 0.0471 | 0.0569 | 0.0313 | 0.0219 | 0.0404 | 0.0160 | 0.0272 | 0.0271 | 0.0330 | 0.0309 | 0.0334 |
| **neg_5** | **L-Phenylalanine** | 0.0002 | 0.0003 | 0.0008 | 0.0007 | 0.0008 | 0.0002 | 0.0003 | 0.0008 | 0.0019 | 0.0022 | 0.0020 | 0.0009 | 0.0007 | 0.0006 | 0.0008 | 0.0024 | 0.0016 | 0.0006 |
| **neg_6** | **Acetyl-DL-Leucine** | 0.0305 | 0.0284 | 0.0261 | 0.0410 | 0.0314 | 0.0279 | 0.0354 | 0.0322 | 0.0474 | 0.0364 | 0.0486 | 0.0472 | 0.0282 | 0.0366 | 0.0404 | 0.0530 | 0.0566 | 0.0316 |
| **neg_7** | **Acamprosate** | 0.0881 | 0.0864 | 0.0594 | 0.0459 | 0.0838 | 0.1370 | 0.0757 | 0.1260 | 0.0350 | 0.0275 | 0.0517 | 0.0389 | 0.0477 | 0.0750 | 0.0404 | 0.0676 | 0.0612 | 0.0801 |
| **neg_8** | **2,4-Dinitrotoluene** | 0.0010 | 0.0008 | 0.0112 | 0.0002 | 0.0027 | 0.0004 | 0.0003 | 0.0009 | 0.0004 | 0.0002 | 0.0004 | 0.0006 | 0.0000 | 0.0003 | 0.0005 | 0.0001 | 0.0001 | 0.0005 |
| **neg_9** | **Saccharin** | 0.0743 | 0.0942 | 0.0931 | 0.0462 | 0.0657 | 0.0623 | 0.0623 | 0.0927 | 0.0413 | 0.0316 | 0.0384 | 0.0370 | 0.0224 | 0.0372 | 0.0143 | 0.0354 | 0.0252 | 0.1064 |
| **neg_10** | **L-Iditol** | 0.0026 | 0.0024 | 0.0020 | 0.0024 | 0.0060 | 0.0027 | 0.0030 | 0.0026 | 0.0031 | 0.0061 | 0.0062 | 0.0065 | 0.0046 | 0.0038 | 0.0082 | 0.0203 | 0.0079 | 0.0035 |
| **neg_11** | **N-Acetyl-L-tyrosine** | 0.0019 | 0.0019 | 0.0037 | 0.0036 | 0.0033 | 0.0028 | 0.0029 | 0.0024 | 0.0044 | 0.0063 | 0.0087 | 0.0044 | 0.0027 | 0.0040 | 0.0029 | 0.0084 | 0.0068 | 0.0040 |
| **neg_12** | **L-Cystine** | 0.0143 | 0.0104 | 0.0268 | 0.0056 | 0.0098 | 0.0273 | 0.0089 | 0.0133 | 0.0071 | 0.0030 | 0.0035 | 0.0070 | 0.0038 | 0.0064 | 0.0065 | 0.0091 | 0.0028 | 0.0137 |
| **neg_13** | **2-Hydroxyadenine** | 0.0002 | 0.0009 | 0.0000 | 0.0010 | 0.0000 | 0.0005 | 0.0001 | 0.0004 | 0.0011 | 0.0022 | 0.0007 | 0.0026 | 0.0040 | 0.0003 | 0.0045 | 0.0056 | 0.0004 | 0.0009 |
| **neg_14** | **Eicosapentaenoic acid** | 0.0045 | 0.0046 | 0.0045 | 0.0038 | 0.0048 | 0.0052 | 0.0168 | 0.0038 | 0.0023 | 0.0042 | 0.0041 | 0.0036 | 0.0021 | 0.0045 | 0.0034 | 0.0041 | 0.0026 | 0.0030 |
| **neg_15** | **N-Acetylneuraminic acid** | 0.0043 | 0.0022 | 0.0039 | 0.0028 | 0.0068 | 0.0057 | 0.0042 | 0.0035 | 0.0022 | 0.0030 | 0.0039 | 0.0024 | 0.0023 | 0.0027 | 0.0016 | 0.0024 | 0.0030 | 0.0042 |
| **neg_16** | **D-Fructose 1,6-bisphosphate** | 0.0017 | 0.0002 | 0.0013 | 0.0012 | 0.0012 | 0.0005 | 0.0022 | 0.0017 | 0.0010 | 0.0023 | 0.0045 | 0.0023 | 0.0045 | 0.0015 | 0.0053 | 0.0044 | 0.0031 | 0.0014 |
| **neg_17** | **Nicolsamide** | 0.0009 | 0.0004 | 0.0015 | 0.0009 | 0.0014 | 0.0008 | 0.0015 | 0.0012 | 0.0010 | 0.0015 | 0.0017 | 0.0017 | 0.0021 | 0.0015 | 0.0014 | 0.0016 | 0.0016 | 0.0008 |
| **neg_18** | **1-Methylguanosine** | 0.0011 | 0.0011 | 0.0012 | 0.0010 | 0.0013 | 0.0011 | 0.0010 | 0.0012 | 0.0005 | 0.0008 | 0.0008 | 0.0008 | 0.0002 | 0.0008 | 0.0005 | 0.0008 | 0.0008 | 0.0012 |
| **neg_19** | **Amygdalin** | 0.0013 | 0.0009 | 0.0009 | 0.0011 | 0.0013 | 0.0015 | 0.0008 | 0.0011 | 0.0006 | 0.0005 | 0.0007 | 0.0006 | 0.0004 | 0.0008 | 0.0007 | 0.0011 | 0.0007 | 0.0012 |
| **neg_20** | **Glycodeoxycholic acid** | 0.0025 | 0.0029 | 0.0057 | 0.0051 | 0.0083 | 0.0046 | 0.0069 | 0.0041 | 0.0008 | 0.0016 | 0.0035 | 0.0020 | 0.0012 | 0.0029 | 0.0013 | 0.0037 | 0.0016 | 0.0109 |
| **neg_21** | **Biliverdin** | 0.0002 | 0.0001 | 0.0005 | 0.0003 | 0.0006 | 0.0002 | 0.0008 | 0.0002 | 0.0002 | 0.0002 | 0.0002 | 0.0002 | 0.0002 | 0.0002 | 0.0001 | 0.0002 | 0.0003 | 0.0002 |
| **neg_22** | **Neohesperidin** | 0.0014 | 0.0000 | 0.0018 | 0.0008 | 0.0011 | 0.0011 | 0.0019 | 0.0017 | 0.0027 | 0.0030 | 0.0024 | 0.0031 | 0.0029 | 0.0025 | 0.0028 | 0.0020 | 0.0023 | 0.0007 |

* Data were calibrated by the peak area of the internal standard.
